# Imaging photoplethysmography reveals differences in the reactions of cerebral and systemic hemodynamics to infusion of vasoactive drugs

**DOI:** 10.3389/fphys.2026.1777457

**Published:** 2026-03-19

**Authors:** Alexey Y. Sokolov, Valeriy V. Zaytsev, Anton V. Shcherbinin, Alexei A. Kamshilin

**Affiliations:** 1 Pavlov Institute of Physiology, Russian Academy of Sciences, St. Petersburg, Russia; 2 Department of Neuropharmacology, Valdman Institute of Pharmacology, Pavlov First St. Petersburg State Medical University, St. Petersburg, Russia; 3 Institute of Automation and Control Processes, Far Eastern Branch of the Russian Academy of Sciences, Vladivostok, Russia; 4 North-Western District Scientific and Clinical Centre, Federal Medical and Biological Agency, St. Petersburg, Russia

**Keywords:** adenosine triphosphate, cerebral perfusion, imaging photoplethysmography, intracranial vessels, norepinephrine, systemic hemodynamic

## Abstract

**Introduction:**

According to the recent data, imaging photoplethysmography has extensive capabilities in clinical and experimental biomedical research. However, relationship between local vasomotor reactions, estimated as fluctuations of the amplitude of pulsatile component (APC) of a photoplethysmographic waveform, and changes in systemic hemodynamic parameters remains unclear. The study aims to assess APC changes concurrently with changes in basic physiological parameters in response to administration of either vasodilating adenosine triphosphate or vasoconstrictor norepinephrine.

**Methods:**

In anesthetized, artificially ventilated rats (n=10), a video recording of the cerebral cortex was performed through thinned parietal bones synchronously with an electrocardiogram. Simultaneously, systemic blood pressure and end-tidal CO_2_ were monitored.

**Results:**

Cerebrovascular effects of vasoactive agents are expressed in transient, pronounced and stereotypical changes in APC for all animals. For both substances, these changes are multiphasic, with at least two episodes of rise and fall in APC that do not correlate with changes in blood pressure. For the first time, opposite reaction vectors of intracranial and systemic hemodynamics were detected with the administration of either vasodilators and vasoconstrictors.

**Discussion:**

Our study demonstrates that APC can be considered as a quantitative marker of local vasodilation/vasoconstriction, which does not necessarily coincide with systemic hemodynamics, thus enabling an experimental investigation of autoregulation processes. The results obtained make it possible to link systemic and intracranial hemodynamic shifts caused by drugs into a single picture and clarify how adenosine triphosphate or norepinephrine affect the cardiovascular system and blood supply to the brain, which is very important due to the widespread use of these drugs in practical medicine.

## 1 Introduction

Over the past few years, various research groups have successfully demonstrated the broad capabilities of imaging photoplethysmography (IPPG), which is a contactless method for assessing perfusion fluctuations *in-vivo* through correlation processing of video frames of biological tissue recorded synchronously with an electrocardiogram, as a helpful tool for clinical and experimental studies, as well as diagnosing. In particular, in abdominal surgery and neurosurgery, IPPG was used for intraoperative visualization and quantitative assessment of changes in blood supply to an organ (Kamshilin et al., 2022; Lai et al., 2022; Shcherbinin et al., 2024), in functional diagnostics – for assessment of cutaneous low-frequency perfusion oscillations (Borik et al., 2022; Mizeva et al., 2025), and in rheumatology – to identify novel instrumental markers of proximal scleroderma (Mamontov et al., 2020a; Zhao et al., 2023). This technique was applied to assess the cerebrovascular functional reserve (Mamontov et al., 2020b; Volynsky et al., 2022) and reaction of the intracranial hemodynamic to somatic and visceral nociception (Lyubashina et al., 2019), to identify optical markers of trigemino-vascular activation (Sokolov et al., 2021), and to reveal duality in response of intracranial vessels to nitroglycerin infusion (Sokolov et al., 2023).

In all of the above-listed studies, the key parameter assessed by the IPPG was the amplitude of the pulsatile component (APC) of an IPPG waveform, changes in which sensitively represented any fluctuations in the tissue perfusion under study (Park et al., 2022). It is worth noting that in most experimental studies of intracranial hemodynamic using IPPG, changes in APC were a consequence of local reactions of perfusion to any stimulus, and these changes were quite often independent of fluctuations in arterial blood pressure (ABP) (Mamontov et al., 2020b; Sokolov et al., 2021, 2023). However, in one study there was a negative correlation between cortical microcirculation assessed by IPPG and changes in systemic ABP (Lyubashina et al., 2019), whereas in another the correlation between changes in ABP and APC was strong and positive (Volynsky et al., 2022). It should be borne in mind that ABP in both these experiments varied, as a rule, within the normal physiological range, and its variations were a consequence of the internal restructuring of systemic hemodynamic in response to either visceral and somatic peripheral stimuli of varying intensity (Lyubashina et al., 2019), or a change in the composition of the inhaled gas mixture (Volynsky et al., 2022). Against the background of these observations, the relationship between changes in cerebral blood supply and marginal fluctuations in systemic blood pressure provoked by vasoactive pharmacological drugs remains unclear, and its study for further in-depth understanding of the nature of the IPPG signal seems interesting and important. In this regard, the first aim of our study was to use IPPG to assess changes in intracranial perfusion in rats induced by harsh pharmacological interventions that severely affect systemic hemodynamic.

Based on the literature data (Boarini et al., 1984; Sollevi et al., 1984; Hussain et al., 2009; Russell et al., 2021), we selected two pharmacological substances leading to an opposite effect on ABP and the cardiac conduction system: adenosine triphosphate (ATP) and norepinephrine (NE) as short-acting hypotensive and hypertensive drugs, respectively. In making this choice, we kept in mind that both ATP and NE are widely used in practical medicine as therapeutic and diagnostic agents (Jentzer and Hollenberg, 2021; Kong et al., 2023; Miyoshi et al., 2023). Therefore, any additional information about their effect on cerebral perfusion may be useful in terms of expanding the understanding of their pharmacodynamics, especially given the existence of conflicting reports on this topic (Froese et al., 2020). Taking these nuances into account, we identified the second aim of our study, which is mirror-related to the first one: to evaluate the cerebrovascular effects of ATP and NE using IPPG.

## 2 Materials and methods

### 2.1 Ethical approval

All experiments were performed in accordance with the ethical guidelines of the International Association for the Study of Pain, the Directive 2010/63/EU of the European Parliament and of the Council on the protection of animals used for scientific purposes, and reported in compliance with the ARRIVE guidelines (https://arriveguidelines.org). The study protocol was approved by the Institutional Animal Care and Use Committee of the Pavlov First St. Petersburg State Medical University (Protocol No. 100_IF2_122023/26_210). Experiments were carried out on adult male Wistar rats (mean body weight 432 ± 27 g, n = 10) that were purchased from the State Breeding Farm “Rappolovo” (Saint Petersburg, Russia) and housed in groups (2–4 per cage) under standard laboratory conditions with food and water available ad libitum. Every effort was made to reduce animal suffering and stress, and to use only the number of experimental subjects necessary to produce reliable data.

### 2.2 Animal preparation

Anesthesia and surgical preparations were performed as described in our previous papers (Mamontov et al., 2020b; Sokolov et al., 2021, 2023; Volynsky et al., 2022). Briefly, the rats were anesthetized by intraperitoneal injection with a mixture of urethane (Sigma, St. Louis, MO, USA) and a-chloralose (Sigma, St. Louis, MO, USA) at an initial dose of 800 mg/kg and 60 mg/kg, respectively. After achieving surgical anesthesia, each rat was placed on a thermostatically controlled heating pad, which provided a constant body temperature during whole experiment. The trachea was intubated for respiratory airflow and end-tidal carbon dioxide measurements. The right femoral artery and vein were cannulated for continuous ABP assessment and drug administration, respectively. The animal’s head was fixed in a stereotaxic apparatus (Stoelting Co., Wood Dale, IL, USA). Closed cranial window (CCW) was formed in each animal by thinning the left and right parietal bones with a micro-drill to the state of a thin membrane, until the intracranial vessels became clearly visible through the remaining intact bone. During the drilling, tissues were cooled using topical application of cold saline. All animals were paralyzed with pipecuronium bromide (Arduan, Gedeon Richter, Budapest, Hungary) at a dose of 1.0 mg/kg initially and then, if necessary, maintained at 0.4 mg/kg. The adequacy of anesthesia was evaluated by the absence of the withdrawal reflex after paw pinch (before myorelaxation) or severe (> 20%) blood pressure fluctuations (after myorelaxation). If necessary, a supplemental dose of anesthetic mixture of urethane/α-chloralose was administered intravenously. After completing the preparation of an animal and before the start of the main part of the experimental measurements, the animal was at rest for at least one hour. After the end of each experiment, the deeply anesthetized rat was sacrificed using a lethal dose of 5% isoflurane.

### 2.3 Study protocol

Each animal (n = 10) was sequentially injected intravenously with four different solutions: ATP (triphosadenine, “Sodium Adenosine Triphosphate,” Ellara, Russia) in two doses of 2 mg/kg and 5 mg/kg, NE (“Noradrenaline”, Aspectus Pharma, Russia) in the dose of 10 μg/kg, and sodium chloride (0.9%) in an equivalent volume (0.8 ml). The order of injections was randomized. Thus, the experiment with any rat included four intervention sessions, each of which lasted 10 min and had a standard sequence of three following epochs: (i) a 30-second pre-infusion period to assess baseline parameters; (ii) infusion of one of the four studied solutions for 45–50 seconds; (iii) a follow-up period during the remaining time. During each 10-min session, we continuously and simultaneously measured main physiological parameters of the animal and the parameter APC reflecting changes in cerebral perfusion. The interval between the end of one session and the beginning of the next was at least 10 minutes, so the total duration of the experiment with one animal was at least 1 hour and 10 minutes.

### 2.4 Measuring techniques

#### 2.4.1 Main physiological parameters

**Figure 1:**
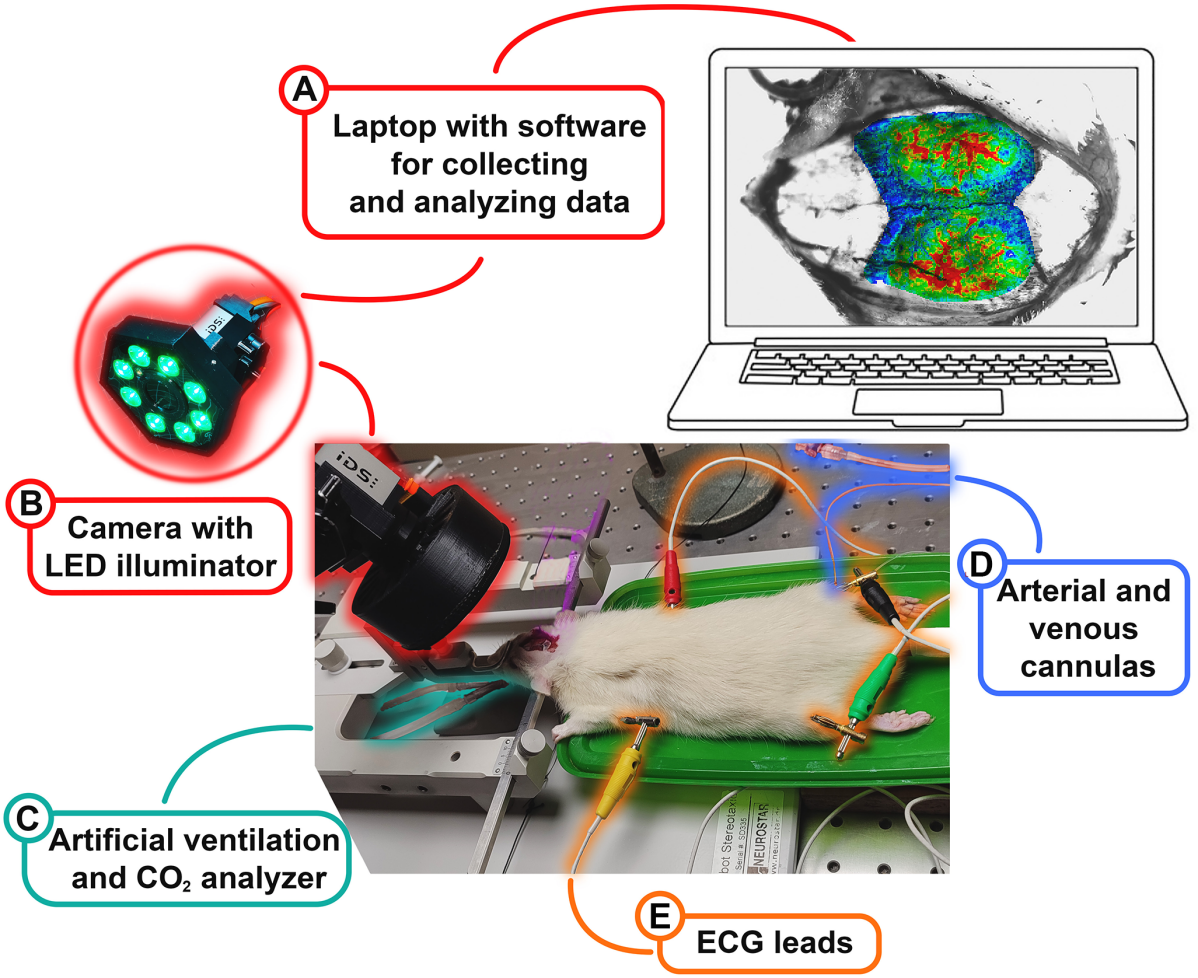
Schematic of experimental setup. It is used for simultaneous measurements of an APC, amplitude of pulsatile component in rat brain, an electrocardiogram, systemic blood pressure, and respiration in response to intravenous administration of various substances. A picture on the laptop screen shows an example of an evaluated APC map superimposed with the image of the rat cortex.

Schematic of experimental setup used to simultaneously measure parameters of cerebral perfusion, systemic hemodynamic, and respiration is shown in **Figure 1**. During the measurements, each animal was under artificial ventilation with room air using a system SAR-830 (CWE, Inc., Ardmore, Pennsylvania, USA), as shown in **Figure 1C**. In all experiments, an electrocardiogram (ECG) was recorded using a digital electrocardiograph operating at a sample rate of 1 kHz (model KAP-01-Kardiotekhnika-EKG, Incart Ltd., Saint Petersburg, Russia). We used a standard three-lead ECG recording with steel needles inserted into the muscle tissue of the rat extremities as electrocardiograph contacts, as seen in **Figure 1E**. Continuous monitoring of ABP and end-tidal CO_2_ was carried out by the pressure sensor (MLT844, AD Instruments Inc., Colorado Springs, USA) and the carbon dioxide analyzer (Capstar-100, CWE, Inc., Ardmore PA, USA), respectively. These data were digitized at a sample frequency of 10 kHz (ADC-DAC Power1401-3, Cambridge Electronic Design, Cambridge, UK) and recorded on the personal computer using Spike2 version 8 software (Cambridge Electronic Design, Cambridge, UK).

#### 2.4.2 Imaging photoplethysmography

The IPPG system used in the study was developed and manufactured in our group. The key (optical) module of the system is a digital camera (IDS GigE Smartek Vision GC1391MP, Imaging Development Systems GmbH, Obersulm, Germany), rigidly mounted together with a light-emitted-diode (LED) illuminator as shown in **Figure 1B**. The illuminator consisted of 8 LEDs (BL-HP20APGCL-5W STAR), operating at the wavelength of 525 ± 30 nm (green light), with a luminous flux of up to 120 lumens, and a power of 5 watts. The LEDs were located around the camera lens (KOWA LM5NCL) to ensure uniform illumination of the area of interest. The camera lens and LEDS were covered with a circular polarizing filter (K&F Concept Nano-X Magnetic CPL) to reduce the influence of specular reflections and motion artifacts, and increase the signal-to-noise ratio. Since this type of polarization filtering does not require any adjustment, it is preferable to the two crossed linearly polarized films that we used in our previous versions of the IPPG system (Kashchenko et al., 2022; Sokolov et al., 2023). Picture of the optical module is shown in **Figure 1B**.

The optical module has been aligned so that the normal to the CCW surface is as close as possible to the optical axis of the camera lens. Such a design of the optical scheme allowed us to minimize the effect of ballistocardiographic artifacts (Moço et al., 2016). To minimize tissue dehydration and increase the transparency of the residual bone for video recording of the rat cerebral cortex, the CCW was covered with mineral oil. The distance between the camera lens and the CCW was about 18 cm. The lens allowed us to focus on the sensor of the camera an image of the rat’s brain sizing approximately 30 by 20 mm. The camera was connected via an Ethernet port to a computer running the Windows 10 operating system. The images were captured using the C^++^ software developed in our group based on the software library supplied by the camera manufacturer. The camera recorded monochrome images in uncompressed (lossless) format with a depth of 8 bits at a rate of 150 frames per second with a spatial resolution of 600 × 400 pixels.

To reveal an optical signal associated with cardiac activity, ECG was recorded simultaneously and synchronously with video frames. To synchronize video and ECG data, we used synchronous pulses generated by the camera at the beginning of each frame. These pulses were continuously received by one of the input channels of the digital electrocardiograph and were digitized in this device in parallel with the signals from the standard ECG leads. By this way, the synchronization accuracy of 1 ms was achieved. Data collection and the management of the entire system were carried out by a personal computer shown in **Figure 1A**.

### 2.5 Data processing

#### 2.5.1 Perfusion index (APC)

**Figure 2:**
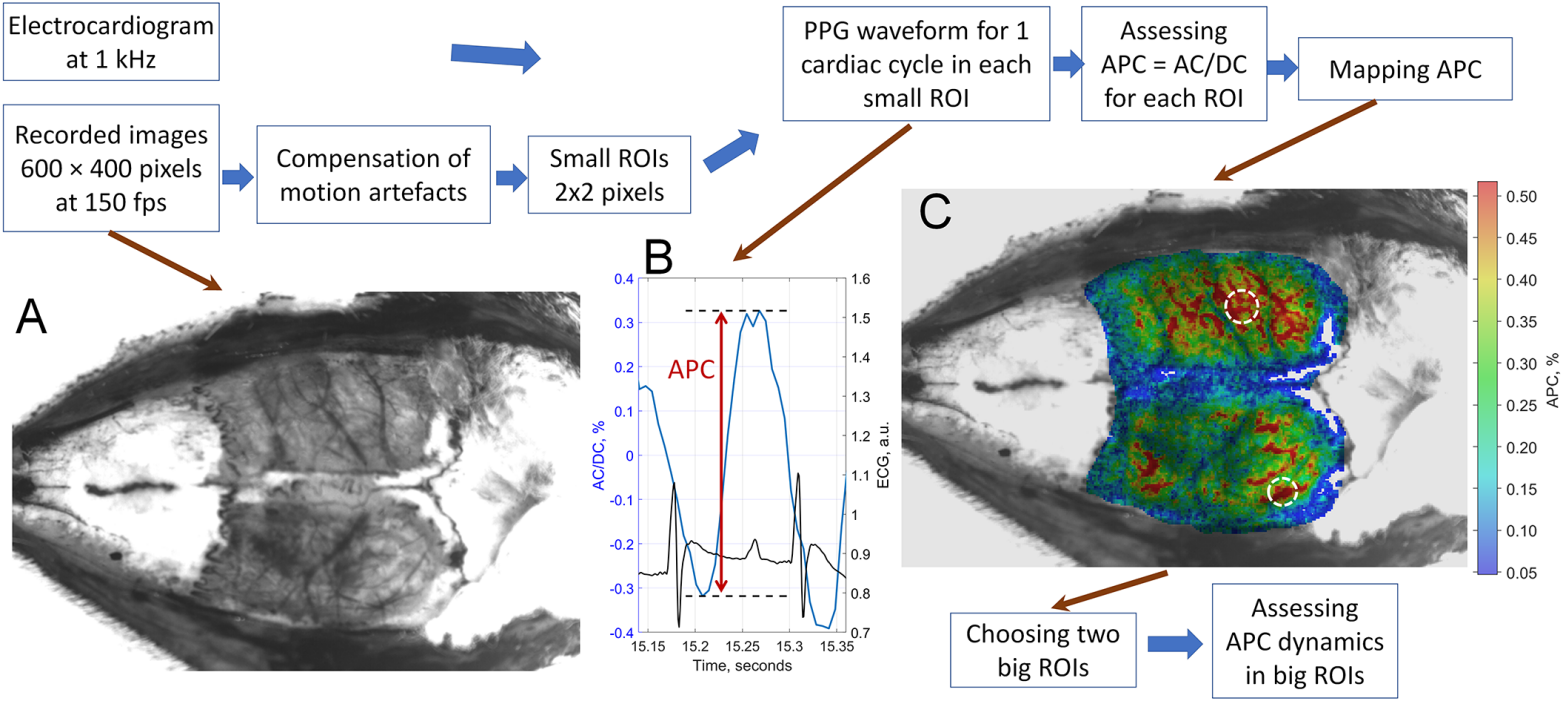
The algorithmic steps of data processing from the ECG-synchronized IPPG system to assess the dynamics of the rat cortex perfusion index. **(A)** An unprocessed image of the rat cerebral cortex through a closed cranial window. **(B)** Frame-by-frame evolution of the average pixel value in a 2×2-pixels ROI after normalization and inversion (blue line) and synchronously recorded ECG signal (black line) with two R-peaks indicating the beginning and end of the selected cardiac cycle. **(C)** An example of a pseudo-colored map of the cortical perfusion index during the selected cardiac cycle overlaid with the unprocessed cortex image. White circles show two big ROIs for estimation the perfusion index response to administration of vasoactive drugs. The color bar on the right shows APC as a percentage.

After recording and saving on the computer, video frames and ECG were processed offline using custom software implemented on the MATLAB^®^ platform (MathWorks Inc., MA, USA) to assess changes in APC, which reflects the perfusion dynamics. Here we used an algorithm that differs from what was used in previous works of our group (Kamshilin et al., 2018; Kashchenko et al., 2022). The main difference is that instead of averaging IPPG signals over several cardiac cycles (in time), averaging over several adjacent regions of interests (ROI), i.e., in space, is used. The main steps of our data processing algorithm are schematically illustrated in **Figure 2**.

At the first stage, a spatial distribution of the APC parameter over rat’s cortex was calculated during just one cardiac cycle, which starts at 15th second of each session (mid of the baseline). The beginning and end of this cardiac cycle was precisely determined using the synchronously recorded ECG. The entire image (see an example of raw image in **Figure 2A**) was divided into ROIs of 2×2 pixels (or 100 × 100 μm in the cortical plane), and the positions of these ROIs were varied from frame to frame to compensate for inevitable motion artifacts using a multi-section image stabilization algorithm described in detail in our previous papers (Kamshilin et al., 2018; Kashchenko et al., 2022). The IPPG waveform was calculated as an evolution of the mean pixel value in each small ROI considering its varying position in each frame. As a rule, the IPPG signal consists of an alternating component (AC, which is fluctuating in accordance with heartbeats) and slowly-varying component (DC). To compensate for uneven illumination, we calculate the AC/DC ratio in each ROI, since both AC and DC components are directly proportional to the incident light intensity (Liu et al., 2014). An example of IPPG waveform estimated in a 2×2 pixels ROI during one cardiac cycle is shown in **Figure 2B**. The difference between the maximum and minimum AC/DC values during the selected cardiac cycle determines the desired APC parameter. Since APC is calculated in each small ROI across the entire field of view of the camera, it allows mapping the spatial distribution of this parameter for the selected cardiac cycle. **Figure 2C** shows a typical APC distribution encoded by pseudo-colors and superimposed on the rat cerebral cortex as seen through CCW. One more example of APC mapping of another rat’s cortex is displayed on the monitor screen of a personal computer shown in **Figure 1A**. One can clearly see that IPPG visualizes the network of arteries. Physiologically, APC indicates changes in the tone of supplying arteries due to their pulsatile nature: the greater the variation in the diameter of the vessel regulated by arterial tone, the higher the amplitude of pulsations, i.e., APC (Lyubashina et al., 2019; Volynsky et al., 2022). Note that our group recently performed a head-to-head comparison of the IPPG system with indocyanine green (ICG) fluorescence angiography during abdominal surgery in the operating room. Quantitative assessment of blood perfusion by IPPG was shown to be in good agreement with data obtained using ICG fluorescence imaging in all surgical cases studied (Kashchenko et al., 2022).

**Figure 3:**
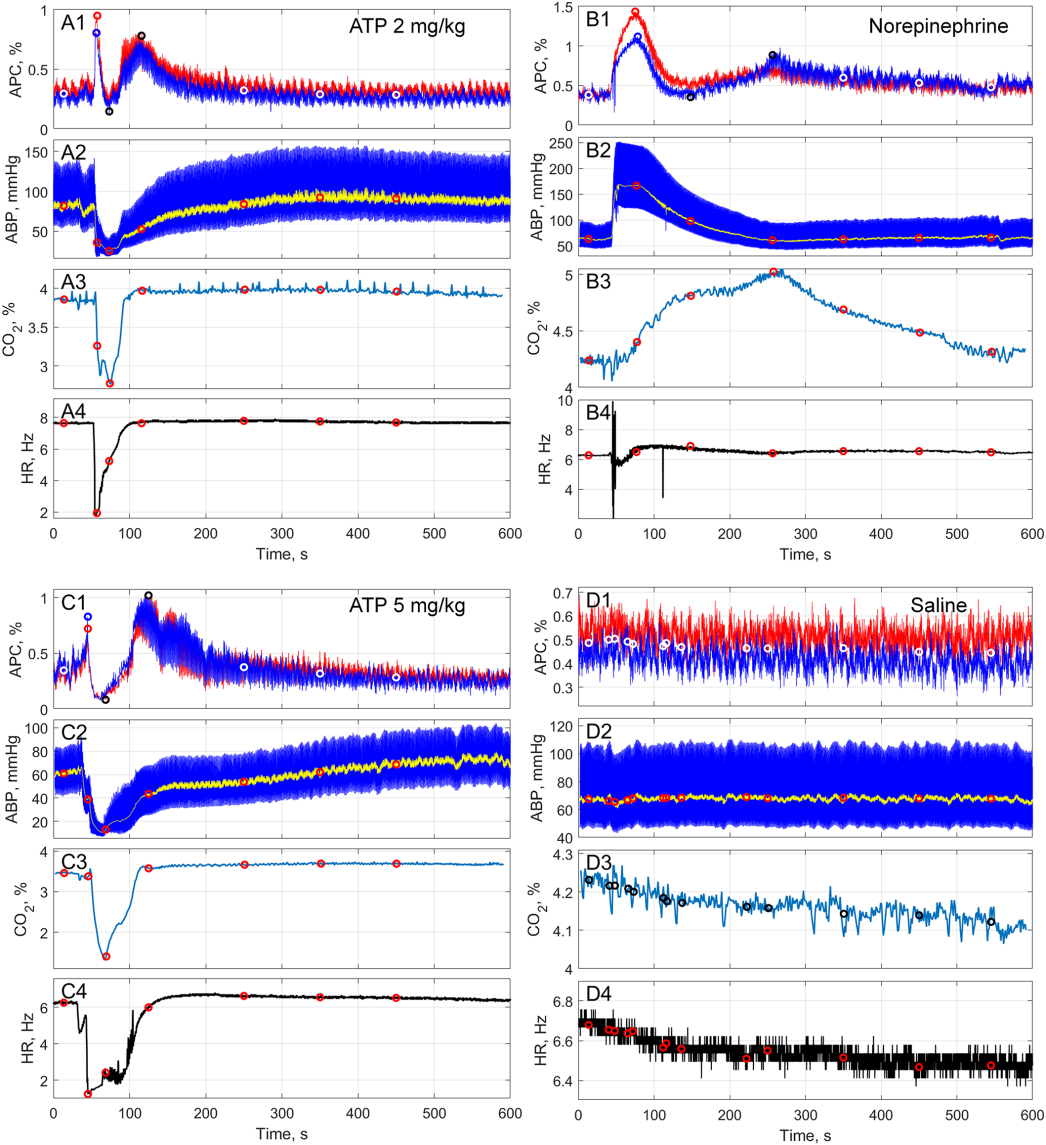
Typical examples of the reaction of APC, ABP, end-tidal CO_2_, and HR to infusion of various substances. **(A)** response to ATP at a dose of 2 mg/kg; **(B)** response to NE at a dose of 10 μg/kg; **(C)** response to ATP at a dose of 5 mg/kg; and **(D)** – response to saline. In each panel, graph X1 shows the dynamics of APC [percent] assessed in the right (red curve) and left (blue curve) hemispheres; graph X2 – the dynamics of ABP [mmHg], in which the yellow curve indicates mean ABP; graph X3 – the dynamics of end-tidal CO_2_ [percent]; and graph X4 – the dynamics of HR [Hz]. Circles on the curves show moments of the onset of key events, determined by the peculiarities of the APC dynamics, namely: baseline, peak, dip, plateau, tail-1, tail-2, and tail-3 (shown from the left to the right). Systemic hemodynamic parameters for the peak, dip, and plateau were assessed at time points averaged over the moments of the corresponding APС events in different hemispheres.

At the second stage, two regions with the higher amplitudes of the pulsatile component were manually selected on the APC map superimposed with the image of cerebral cortex, one in the right and the other in the left hemisphere. An example of the selected big ROIs (white circles) is shown in **Figure 2C**. Each selected region contained from 25 to 55 ROIs. Then we repeated calculations of the AC/DC dynamics as in the previous stage, but did so only for the selected big ROIs and continuously over entire 10-minute session using a floating time-window of duration equal to the mean cardiac cycle to compensate for motion artifacts. A small number of chosen big ROIs was selected to reduce the processing time. These calculations allowed us to assess the APC parameter in each big ROI for every cardiac cycle during the entire 10-minute session. After averaging the APC parameters over the respective ROIs separately for each of the two selected regions, we obtain two resulting dependencies representing changes in the perfusion index in each hemisphere. Examples of the APC evolution are shown in **Figure 3**.

#### 2.5.2 Comparison of responses in different animals

As mentioned above, simultaneously with the assessment of changes in the cortical perfusion index in response to drug infusion, we also measured the dynamics of ABP, end-tidal CO_2_, and heart rate (HR). To compare responses of these parameters to infusion among different animals, we normalized the data for each parameter relative to its corresponding baseline value, taking it to be 100%. The magnitudes ​​of these normalized parameters were expressed as percentages to plot them on a single graph and compare their changes at the same time points. Since changes in APC during 10-minute IPPG recording before and after administration of tested substances (with the exception of saline solution) were stereotypical, it was APC that was chosen as the leading parameter. This allowed us to clearly identify 7 standard points or segments in the time course of APC evolution, in which these changes manifested themselves most brightly, forming certain peaks, dips, and plateaus (see, for example, graphs A1, B1 and C1 in **Figure 3**). It should be noted that the time position of these features differed depending on the injected substance (either ATP or NE) and the dose of administration for ATP. The values of other analyzed parameters (ABP, end-tidal CO_2_, and HR) were assessed at the same time points, which allowed us to reveal the interdependence of their changes during specific periods of the time course. The effect of saline solution was assessed on a composite time course (13 points in total, see the graph D1 in **Figure 3**) consisting of all the time points identified by analyzing the dynamics of the APC signal after the administration of both ATP (in two doses) and NE.

### 2.6 Statistical methods

To assess the significance of differences in the values of the estimated parameters at the identified time points, Two-Way Repeated-Measures ANOVA with Dunnett’s or Sidak’s multiple comparisons tests were used. If the data did not have a normal distribution according to the Shapiro-Wilk criterion, the analysis was performed after its rank transformation. Taking into account the relatively small sample (n = 10), the nonparametric Friedman test was used to compare unranked native data, but Dunn’s multiple comparisons test was not used due to its insufficient power to identify the significance of differences in conditions of very strong multi-vector data fluctuations. If it was necessary to compare two paired samples, the Wilcoxon matched-pairs signed rank test was used. All data were statistically analyzed using GraphPad Prism 8 software (GraphPad Software Inc., San Diego, CA, USA). *P* < 0.05 was defined as statistically significant. To describe the results, the data was expressed as medians with interquartile ranges (Me[Q1–Q3]) or as mean with standard deviation (M ± SD). Boxplots were constructed in the Tukey style.

## 3 Results

### 3.1 Initial state of the studied parameters

Since the infusion of various substances into each animal was carried out sequentially, it was tested to what extent this approach affects the initial values ​​of the APC and every of the three systemic physiological parameters (mean ABP, end-tidal CO_2_, and HR) evaluated before every of the four interventions. No significant difference in baseline values was found for any parameter, as summarized in **Table 1**.

**Table 1:** Baseline values of APC and the systemic physiological parameters under study.

Parameter	ATP, 2 mg/kg (n = 10)	ATP, 5 mg/kg (n = 10)	NE (n = 10)	Saline (n = 10)	Friedman test
APC, %	0.25 [0.18-0.32] ^a)^	0.24 [0.21-0.30]	0.23 [0.16-0.29]	0.26 [0.20-0.32]	*P* = 0.67; *F* = 1.56
Mean ABP, mmHg	65 [58-84]	63 [58-79]	69 [58-81]	66 [55-92]	*P* = 0.22; *F* = 4.44
End-tidal CO_2_, %	3.7 [3.6-3.8]	3.8 [3.6-3.9]	3.7 [3.6-3.9]	3.7 [3.6-3.8]	*P* = 0.95; *F* = 0.36
HR, Hz	6.9 [6.3-7.5]	7.0 [6.4-7.3]	7.0 [6.5-7.4]	6.9 [6.7-7.5]	*P* = 0.81; *F* = 0.96

^a)^ The data is presented as Me[Q1–Q3].

### 3.2 Infusion of ATP

In all animals (n = 10), the administration of ATP in both doses led to a stereotypic response of both APC and other systemic indicators (mean ABP, end-tidal CO_2_, and HR), as shown in **Figures 3A, C**). Analysis of the APC evolution allowed us to identify key events that are regularly observed in the response curves, designated as baseline, peak, dip, plateau, and three points in the rest of the curve (tail-1, tail-2, and tail-3). In these events, APC magnitudes were calculated as follows: peak and plateau – maximum values, dip – minimum value, baseline – average value over the interval of 0–27 seconds, and for the relaxation - average values over the intervals of 200-300, 300–400 and 400–500 seconds. The time points (average over animals) at which the peak, dip, and plateau occur are summarized in **Table 2**. Since for every event on the APC curve a certain magnitude of the systemic parameters (median ABP, end-tidal CO_2_, and HR) was observed at the same time from the beginning of the session, a time-course of changes in the estimated parameters was prepared by normalizing each parameter in the key-point to its baseline value. The time-course was universal for all the animals, however, the position on the time scale of the three key-events (peak, dip, and plateau) depended on which drug was administered, and on the dose of ATP administration, as well (see **Table 2**, **Supplementary Materials**).

**Table 2:** The time of occurrence of the key events after infusion of different drugs.

Substance	dose [μg/kg]	Peak [sec]	Dip [sec]	Plateau [sec]	Tail-1 [sec]	Tail-2 [sec]	Tail-3 [sec]
ATP	2000	48 ± 4^a^	65.0 ± 7.5	116 ± 14	200 – 300^b^	300 – 400	400 – 500
ATP	5000	40 ± 5	64.5 ± 5.0	137 ± 26	200 – 300	300 – 400	400 – 500
NE	10	72 ± 11	112 ± 15	222 ± 27	300 – 400	400 – 500	500 – 590

^a^ Time is presented as (M ± SD) from the beginning of the session; ^b^ the time interval for averaging parameters is shown.

**Figure 4:**
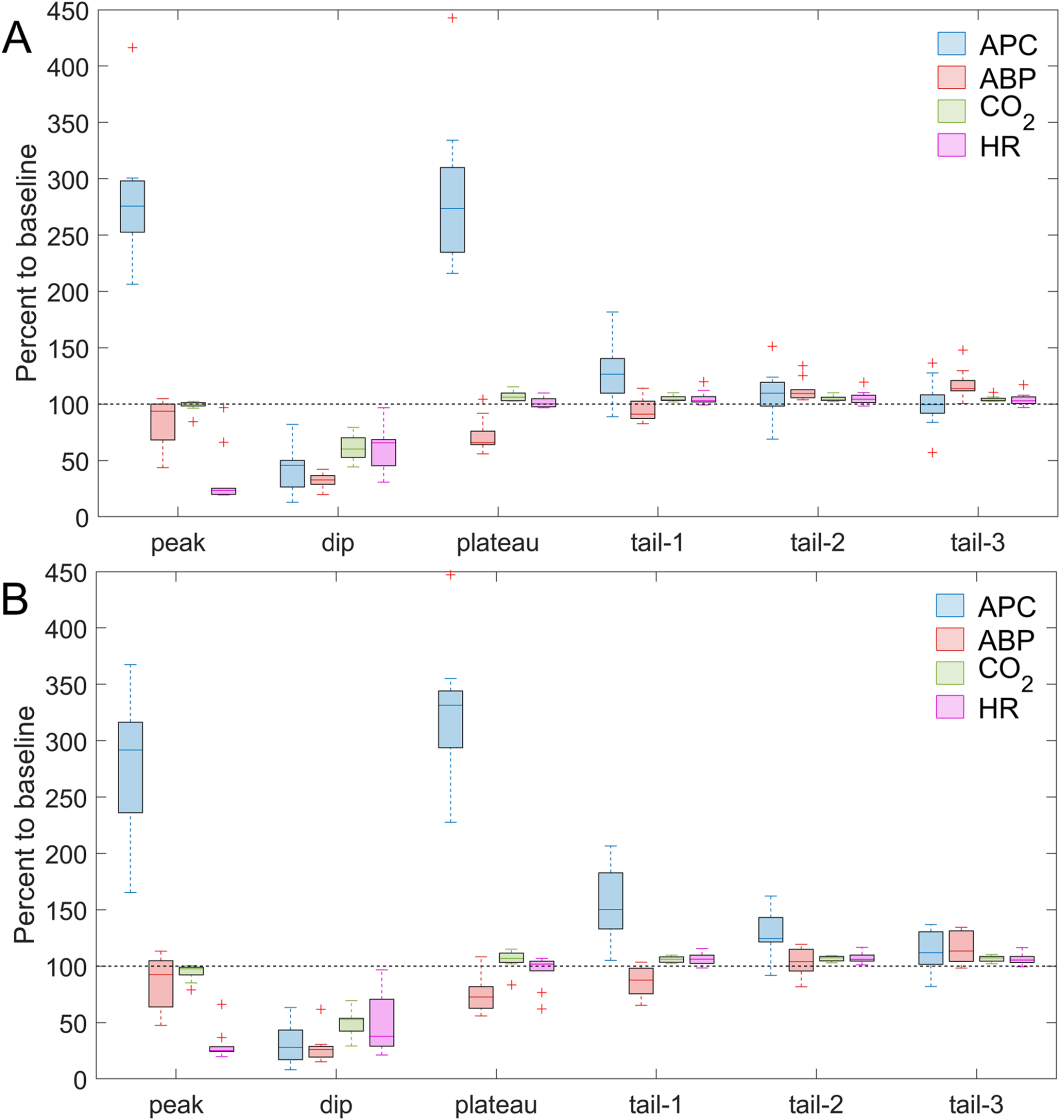
Comparison of the changes in APC and systemic physiological parameters caused by ATP infusion. The data are normalized to their respective baseline values and assessed in the six identified time-points for the key events due to administration of ATP in the dose of 2 mg/kg **(A)** and 5 mg/kg **(B)**. The data is shown as Me[Q1-Q3] measured for the whole sample (n = 10), and statistical significance in differences is given in the text and in **Table 3**. Blue, red, green, and magenta boxes in both panels show the data of APC, ABP, CO_2_, and HR, respectively.

As one can see in **Figures 3A, C** the administration of ATP at both doses led to significant changes in both cortical perfusion (indexed by APC) and systemic physiological parameters. For infusion of ATP at 2 mg/kg, the two-way ANOVA showed significant effects of Event-in-time: *F*(2.188, 78.78) = 103.6, *P* < 0.0001, and Event-in-time × Parameters: *F*(18, 216) = 82.23, *P* < 0.0001. For ATP at 5 mg/kg, there were also significant effects of Event-in-time: *F*(2.784, 100.2) = 134.3, *P* < 0.0001, and Event-in-time × Parameters: *F*(18, 216) = 82.23, *P* < 0.0001. This dramatic response was found in all animals under study. Magnitudes of these parameters for every key event including the statistical significance of differences compared to the respective baselines are summarized in **Table 3**. **Figure 4** shows the dynamics of the normalized parameters in response to ATP infusion in both doses.

**Table 3:** Dynamics of APC, mean ABP, end-tidal CO_2_, and HR when ATP is administrated.

Parameter	ATP dose, mg/kg	baseline	peak	dip	plateau	tail-1	tail-2	tail-3
APC, [%]	2	0.25^a^ [0.18-0.32]	0.66 ****^b^ [0.52-0.90]	0.12 **** [0.05-0.17]	0.68 **** [0.52-1.02]	0.32 [0.21-0.38]	0.26 [0.19-0.32]	0.23 [0.18-0.30]
	5	0.24 [0.21-0.30]	0.76 **** [0.56-0.79]	0.07 **** [0.03-0.13]	0.84 **** [0.65-0.95]	0.37 ** [0.28-0.46]	0.33 * [0.25-0.36]	0.29 [0.23-0.33]
Mean ABP, [mmHg]	2	65 [58-84]	56 [47-66]	21 **** [19-25]	46 ** [42-54]	61 [53-79]	73 * [63-89]	80 * [68-89]
	5	63 [58-79]	57 [38-70]	16 **** [13-22]	48 ** [43-54]	54 [47-70]	66 [56-86]	74 * [67-88]
End-tidal CO_2_, [%]	2	3.7 [3.6-3.8]	3.6 [3.5-3.8]	2.3 **** [1.9-2.6]	4.0 ** [3.8-4.1]	3.9 *** [3.8-4.0]	3.9 *** [3.7-4.0]	3.9 *** [3.7-4.0]
	5	3.8 [3.6-3.9]	3.5 [3.3-3.8]	2.0 **** [1.6-2.1]	4.2 [3.5-4.3]	4.1 *** [3.7-4.2]	4.1 **** [3.7-4.2]	4.1 *** [3.7-4.3]
HR, [Hz]	2	6.9 [6.3-7.5]	1.6 *** [1.4-2.5]	4.2 *** [3.2-5.3]	7.0 [6.6-7.3]	7.3 [6.9-7.7]	7.4 [6.9-7.7]	7.3 [6.7-7.6]
	5	7.0 [6.4-7.3]	1.8 ***** [1.7-2.0]	2.4 ** [2.0-5.5]	6.9 [5.9-7.4]	7.4 * [7.0-7.6]	7.5 ** [7.0-7.7]	7.5 * [6.9-7.6]

^a^ The data is shown as Me[Q1–Q3].

^b^ The statistical significance of difference in respect to the baseline denoted by *, **, *** and **** corresponds to *P* < 0.05, *P* < 0.01, *P* < 0.001, and *P* < 0.0001, respectively, Dunnett’s multiple comparisons test.

When comparing the dynamics of changes in APC, ABP, end-tidal CO_2_, and HR at key points of the time course (panel A and panel C in **Figures 3**, **4**, **Table 3**), it is noteworthy that at the peak point, an explosive escalation of APC (up to 279[252-302] % at the ATP dose of 2 mg/kg and to 292[233-316] % at 5 mg/kg, both in respect to the baseline) occurs against the background of a clear (although not yet statistically significant) decrease in ABP. Such a decrease is accompanied by an extreme bradycardia (drop in HR down to 23[20-35] % at the ATP dose of 2 mg/kg and to 25[24-31] % at 5 mg/kg, both in respect to the baseline), which emphasizes the occurrence of diametrically opposite vectors of reaction of intracranial and systemic hemodynamic to the ATP administration. At the dip point, the indicators of all assessed parameters are at their minimum level, whereas at the plateau point there is only a take-off of APC (up to 275[233-326] % at the ATP dose of 2 mg/kg and to 331[289-347] % at 5 mg/kg), which is significant compared to the baseline but smoother compared to the peak. At the same time, the increase in APC in the plateau is observed simultaneously with a significantly low mean ABP (down to 66[63-80] % at the ATP dose of 2 mg/kg and to 73[62-83] % at 5 mg/kg) compared to the baseline. It means that the fact of the counter directed response in cerebral perfusion and systemic ABP is again revealed. In general, all the main dramatic events in the dynamics of the assessed parameters occur in the first 200 seconds of recording, after which their relatively rapid recovery begins, which is noticeable in the trajectories of the recorded signals at the points tail-1 through tail-3.

Compared with the action of ATP at a dose of 2 mg/kg, the effect of this substance at a dose of 5 mg/kg on the APC dynamics was significantly more pronounced at the points of dip, plateau, tail-2, and tail-3: *P* = 0.04, *P* = 0.02, *P* = 0.03, and *P* = 0.01, respectively (Wilcoxon matched-pairs signed rank test for the normalized data). Moreover, when ATP was administered at a dose of 5 mg/kg, the peak occurred earlier (40 ± 5 vs 48 ± 4 s), and the plateau – later (137 ± 26 vs 116 ± 14 s) compared with ATP infusion at 2 mg/kg; *P* = 0.004 and *P* = 0.01, respectively, Wilcoxon matched-pairs signed rank test (see **Table 2**). These observations indicate a longer phase of the dip (compare graphs A1 and C1 in **Figure 2**). Finally, APC was restored more quickly to the baseline level after administration of ATP at the dose of 2 mg/kg: a statistically significant difference compared to baseline disappeared already at the time-point tail-1, whereas at the dose of 5 mg/kg this disappearance was observed at the tail-3, which is 200 seconds later. Of all the systemic indicators, only the decrease in the end-tidal CO_2_ was more significant after ATP infusion at the dose of 5 mg/kg compared with that at 2 mg/kg (*Р* = 0.03, Wilcoxon matched-pairs signed rank test). No significant difference was revealed between the responses of either ABP or HR to the administration of ATP in different doses at any of the key points of the time course, although at the dip point there was a tendency to a more pronounced decrease in both ABP and HR with an increase in the dose of ATP. Responses of cortical and systemic hemodynamic parameters to ATP infusion are shown for all studied animals in **Supplementary Figures 1**-**S5** (dose 2 mg/kg) and in **Supplementary Figures 6**-**S10** (dose 5 mg/kg) in **Supplementary Materials**.

### 3.3 Infusion of norepinephrine

**Figure 5:**
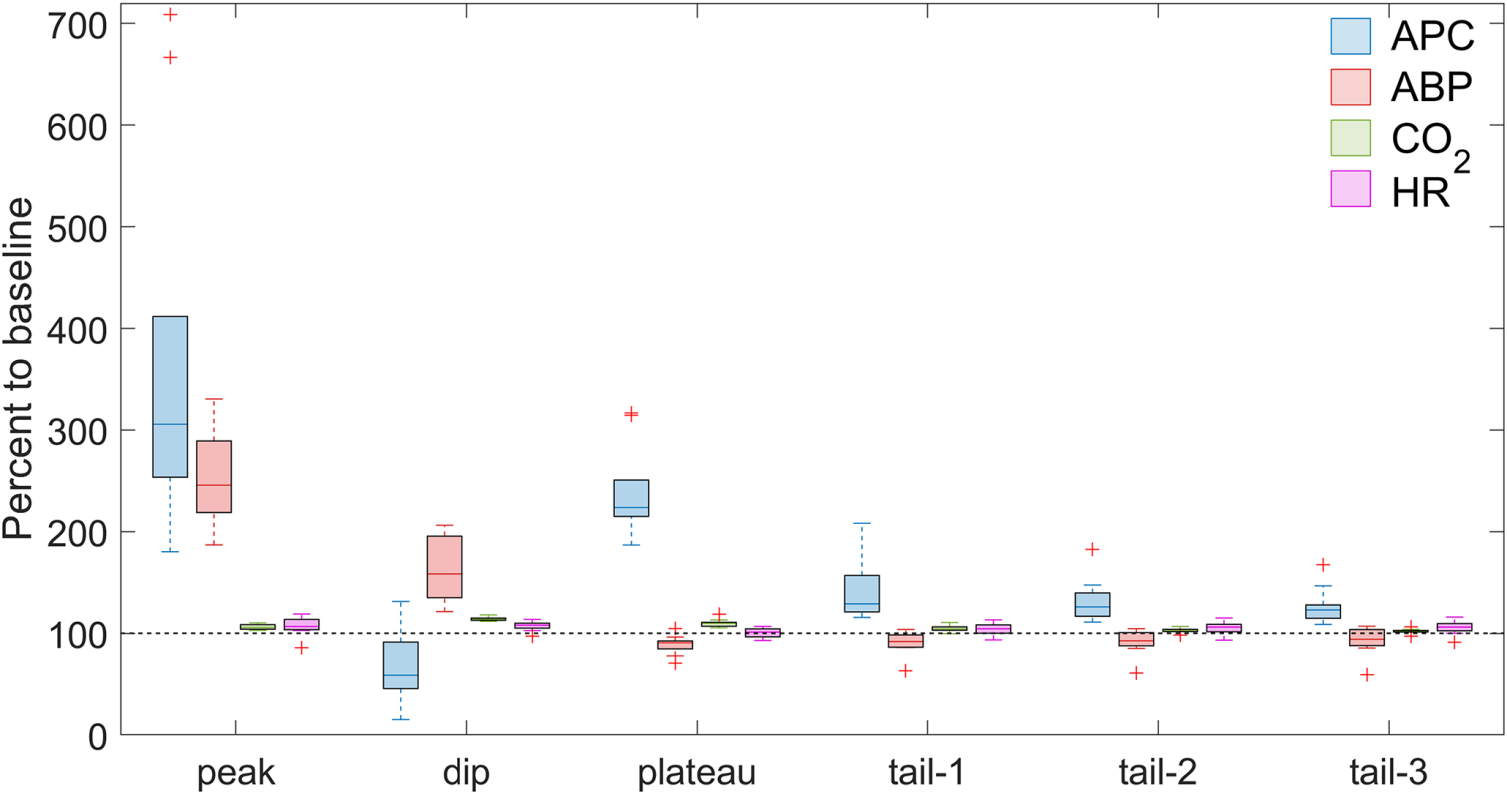
Comparison of the changes in APC and systemic physiological parameters due to NE infusion. Parameters are normalized to their respective baseline values and assessed in the six identified time-points for the key events due to administration of NE in the dose of 10 μg/kg. The data is shown as Me[Q1-Q3] measured for the whole sample (n = 10), and statistical significance in differences is shown in the text and in **Table 4**. Blue, red, green, and magenta boxes represent APC, ABP, CO_2_, and HR data, respectively.

As with ATP administration, all animals (n = 10) had a stereotypic response of cortical perfusion (parameter APC), parameters of the systemic hemodynamic, and end-tidal CO_2_ to the NE infusion at a dose of 10 μg/kg, a typical example is shown in **Figure 3B**. However, there is a clear difference between reactions to ATP and NE (compare panel A or C vs panel B in **Figure 3**). In the evolution of APC, we observe the same sequence of key events (baseline, peak, dip, plateau, and so on) as with the infusion of ATP, but all the key events in the case of NE occur much later than in the case of ATP (see **Table 2**). At the same time, the pattern of changes in systemic parameters after NE infusion was radically different from that after ATP administration (see **Figure 3**). Again, for the infusion of NE, the two-way ANOVA showed significant effects of Event-in-time: *F*(1.143, 41.46) = 50.52, *P* < 0.0001, and Event-in-time × Parameters: *F*(18, 216) = 25.73, *P* < 0.0001. The magnitude of both APC and systemic hemodynamic parameters for every key event including the statistical significance of differences in respect the respective baselines are summarized in **Table 4**. By analogy with ATP, to demonstrate comparative response of the parameters to the administration of NE, we prepared the time course of changes in the parameters assessed in key events and normalized to the corresponding baseline values, which is shown in **Figure 5**.

**Table 4:** Dynamics of APC, mean ABP, end-tidal CO_2_, and HR in the case of NE administration.

Parameter	baseline	peak	dip	plateau	tail-1	tail-2	tail-3
APC, [%]	0.23 ^a^ [0.16-0.29]	0.77 **^b^ [0.57-1.00]	0.13 * [0.12-0.19]	0.52 **** [0.43-0.67]	0.32 ** [0.25-0.37]	0.29 ** [0.24-0.36]	0.28 ** [0.23-0.35]
Mean ABP, [mmHg]	69 [58-81]	170 **** [162-180]	115 *** [100-121]	59 * [51-73]	58 [51-79]	60 [51-80]	60 [52-79]
End-tidal CO_2_, [%]	3.7 [3.6-3.9]	4.0 *** [3.8-4.1]	4.2 **** [4.1-4.5]	4.1 ** [3.9-4.3]	3.9 ** [3.7-4.1]	3.8 [3.7-4.0]	3.8 [3.6-4.0]
HR, [Hz]	7.0 [6.5-7.4]	7.5 [6.8-7.9]	7.4 ** [7.1-7.9]	6.8 [6.6-7.3]	7.4 [6.6-7.4]	7.5 [6.6-7.6]	7.5 [6.6-7.7]

^a^ The data is shown as Me[Q1–Q3].

^b^ The statistical significance of difference in respect to the baseline denoted by *, **, *** and **** corresponds to *P* < 0.05, *P* < 0.01, *P* < 0.001, and *P* < 0.0001, respectively, Dunnett’s multiple comparisons test.

When comparing the dynamics of changes in APC, mean ABP, end-tidal CO_2_, and HR assessed at key events (**Figures 3B**, **5**, **Table 4**), it is noteworthy that the maximal cortical perfusion (APC = 306[248-475] % in respect to the baseline) corresponds to the powerful hypertensive reaction (mean ABP = 246[214-290] % in respect to the baseline). It means a unidirectional response to NE of the intracranial perfusion and the cardiovascular system as a whole. In contrast, in the dip point, the perfusion index falls significantly below the baseline (59[45-92] %), while the mean ABP remains at a significantly higher level compared to the baseline (158[134-197] %), i.e., at this key event, diametrically opposed vectors of reactions in local and systemic hemodynamic are observed. In the plateau event, the vectors of changes in APC and ABP are again reversed: APC significantly increases (224[214-267] % compared to the baseline), and mean ABP falls significantly below the baseline (91[83-93] %). Then, in the relaxation phase (tail-1 through tail-3), mean ABP remains reduced (although not significantly) compared to the baseline, while APC is steadily and significantly higher than in the baseline. To summarize, all the main dramatic changes in the assessed parameters occur within approximately 300 sec after NE infusion, which is 1.5 times longer than the duration of the most noticeable hemodynamic reactions to ATP administration. Responses of cortical and systemic hemodynamic parameters to NE infusion are shown for all studied animals in **Supplementary Figures 11**-**S15** in **Supplementary Materials**.

### 3.4 Effect of saline infusion

**Figure 6:**
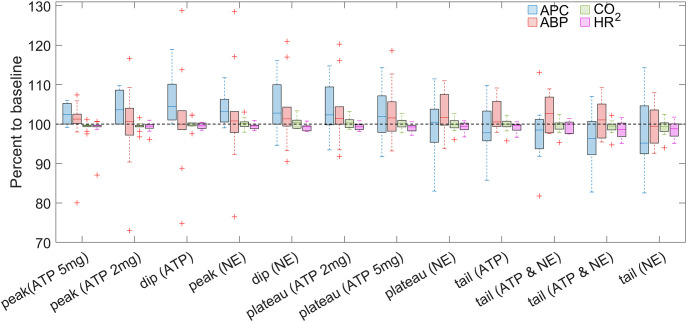
Changes in the measured parameters in response to saline administration. Comparison of the changes in APC, systemic physiological parameters, and end-tidal CO_2_ normalized to their respective baseline values and assessed in 12 identified time-points for the key events in the case of saline administration. The data is shown as Me[Q1-Q3] measured for the whole sample (n = 10). Blue, red, green, and magenta boxes represent APC, ABP, CO_2_, and HR data, respectively.

Since the moment of occurrence of every key event in cortical perfusion caused by drug infusion depends on the type of drug and the dose of ATP (see **Table 2**), to carefully assess the effect of saline infusion, we evaluated the normalized (relative to baseline) changes in hemodynamic parameters at 13 time-points fused for all cases and identified above. Although the RM two-wave ANOVA revealed significant differences in the data array when analyzing the whole massive of the parameters in response to saline infusion (Event-in-time: *F*(2.928, 105.1) = 3.058, *P* = 0.0328, and Event-in-time × Parameters: *F*(36, 432) = 2.037, *P* = 0.0005), after pairwise comparisons of each parameter normalized to the corresponding baseline level, no significant differences were found at every key event: all *P* > 0.06, Dunnett’s multiple comparisons test. An example of changes in APC, mean ABP, end-tidal CO_2_, and HR in response to saline infusion is shown in panel D in **Figure 3**, while the parameters averaged over all rats are shown in **Table 5**. As seen, all the parameters remain stable during the measurement session. Such a stability in the case of saline infusion is observed for all animals under study, as shown in **Figure 6**, which presents the comparative dynamics of hemodynamic parameters normalized to the corresponding baseline level. Responses of cortical and systemic hemodynamic parameters to saline infusion are shown for all studied animals in **Supplementary Figures 16**-**S20** in **Supplementary Materials**.

**Table 5:** Dynamics of APC, mean ABP, end-tidal CO_2_, and HR in the case of saline infusion.

Parameter	baseline	peak ATP-5	peak ATP-2	dip ATP	peak NE	dip NE	plateau ATP-2
APC, [%]	0.26 [0.20-0.32] ^a^	0.27 [0.21-0.33]	0.27 [0.22-0.34]	0.27 [0.22-0.35]	0.27 [0.22-0.35]	0.27 [0.21-0.35]	0.26 [0.21-0.35]
mean ABP, [mmHg]	66 [55-92]	66 [58-82]	65 [58-81]	68 [58-81]	69 [60-81]	67 [63-84]	67 [63-85]
End-tidal CO_2_, [%]	3.7 [3.6-3.9]	3.7 [3.5-3.9]	3.7 [3.5-3.9]	3.8 [3.6-4.0]	3.8 [3.6-4.0]	3.8 [3.6-4.0]	3.8 [3.6-4.0]
HR, [Hz]	6.9 [6.7-7.5]	6.8 [6.6-7.4]	6.9 [6.7-7.4]	6.9 [6.7-7.4]	6.9 [6.6-7.4]	6.9 [6.6-7.5]	6.9 [6.6-7.5]
Parameter	plateau ATP-5	plateau NE	tail (ATP)	tail (ATP & NE)	tail (ATP & NE)	tail (NE)
APC, [%]	0.26 [0.21-0.35]	0.24 [0.21-0.34]	0.24 [0.21-0.33]	0.24 [0.19-0.33]	0.24 [0.20-0.31]	0.25 [0.20-0.3]
mean ABP, [mmHg]	67 [61-86]	68 [58-89]	68 [57-92]	68 [58-95]	68 [57-91]	68 [56-88]
End-tidal CO_2_, [%]	3.8 [3.6-3.9]	3.8 [3.5-3.9]	3.8 [3.6-3.9]	3.8 [3.6-3.9]	3.8 [3.5-3.8]	3.7 [3.5-3.8]
HR, [Hz]	6.9 [6.5-7.5]	6.9 [6.5-7.5]	6.9 [6.5-7.5]	7.0 [6.5-7.5]	6.9 [6.4-7.4]	6.9 [6.4-7.4]

^a^ The data is shown as Me[Q1–Q3].

## 4 Discussion

Our study has shown that in contrast to saline, bolus intravenous administration of both ATP and NE leads to reversible, multidirectional, and dramatic shifts in systemic hemodynamic, and causes complex cerebrovascular reactions that are closely associated with these shifts. The high sensitivity and informativeness of the ECG-synchronized IPPG system applied in this work to assess the cerebrovascular response to vasoactive drug infusion should be emphasized. It is worth noting that the measurements were carried out when the cerebral cortex was illuminated with green light. This light is effectively absorbed by red blood cells (RBCs), which determines its shallow penetration depth of 0.3 mm or less (Anderson and Parrish, 1981), making the probability of its interaction with pulsating arteries very low. However, it has been many times reported that it is when green light illuminates biological tissue *in vivo* that the greatest modulation of its intensity at heart rate is observed (Verkruysse et al., 2008; Maeda et al., 2011; Fallow et al., 2013). Given the known facts that capillaries do not pulsate and that their average diameter is smaller than the average size of RBCs (Fung et al., 1966), we are forced to state that the widely accepted model of PPG signal formation, which says that periodical fluctuations in blood volume in arteries modulate the amount of absorbed photons, the so-called blood volume model (Allen, 2007), contradicts these experimental observations. To resolve this contradiction, our team proposed an alternative (tissue compression) model for PPG waveform generation in which the main cause of light modulation is considered to be mechanical compression and relaxation of the intercapillary tissue by the pulsating walls of nearby situated arteries and arterioles (Kamshilin et al., 2015). A decrease in vascular tone in feeding arteries is accompanied by an increase in APC, since the soft, pliable vessel’s wall is able to stretch with greater amplitude in response to blood pressure pulses. Conversely, an increase in tone (i.e., an increase in wall tension due to vasoconstriction) should lead to a decrease in APC. It is worth noting that pulsations occur in both constricted and dilated arteries, the difference is only in the amplitude of these pulsations, which is definitely lower for constricted vessels. Therefore, the pulsatile component of the PPG waveform is synchronized with the arrived pulse wave and is related to changes in blood volume supplied by the artery (Park et al., 2022). Under normal conditions (normovolemia and the absence of sudden fluctuations in blood pressure), the larger the diameter of the arteries, the more blood they supply to the tissue per unit time, which is commonly interpreted as greater perfusion. In turn, the wider the vessel, the more pliable its walls (lower tone) and larger the amplitude of their pulsations. By this way, the capillary network serves as a naturally distributed transducer of the pulse waves of arterial blood pressure into changes in light intensity (Kamshilin et al., 2015, 2025). The closer the pulsating artery is located to the cerebral cortex, the greater the amplitude of the modulation of the reflected light intensity will be observed. Therefore, our optical system still evaluates changes in the tone of deeply located arteries, although the green light does not directly interact with them.

ATP is a purine nucleotide, an intracellular messenger and energy substrate, neurotransmitter and nucleic acid precursor, also known as a pharmacological drug used in clinical practice for therapeutic and diagnostic purposes. Exogenous ATP is positioned mainly as a nonselective vasodilator, which in humans and animals exhibits its relaxation properties in relation to vessels of various localization, such as coronary arteries (Somló, 1955; Kitakaze et al., 1999; Pivovarova et al., 2022; Kong et al., 2023), superior mesenteric arteries (Watanabe et al., 2016), pial arteries (Forrester et al., 1979), major cerebral vessels (Hussain et al., 2009), and brachial artery (Shepherd et al., 2016). Intravenous administration of ATP has a hypotensive effect in humans (Hussain et al., 2009), dogs (Boarini et al., 1984; Sollevi et al., 1984), monkeys (Van Aken et al., 1984), and rats (Delbro and Burnstock, 1987; Saano et al., 1990), which is consistent with its vasodilating activity. It was shown in anesthesiologic practice that “bolus injection of ATP is a simple, practical and effective method for attenuating the hypertensive response to laryngoscopy and tracheal intubation” (Mikawa et al., 1991). At the same time, ATP, being, along with neuropeptide Y, a sympathetic co-transmitter of noradrenaline (Racchi et al., 1999; Huidobro-Toro and Donoso, 2004; Li et al., 2023; Sohn and Jenei-Lanzl, 2023), can cause vasoconstriction, as, for example, it was demonstrated in a preparation of human saphenous vein (Racchi et al., 1999). There is a lot of information about the dose-dependent biphasic action of ATP, when dilatation is followed by constriction and vice versa, for example, in isolated rat mesenteric small arteries (Juul et al., 1993), isolated rat aortic rings (Dominiczak et al., 1991), rat cerebral penetrating arterioles (Dietrich et al., 2009), feline pulmonary vascular bed (Neely et al., 1989), and rat intrapulmonary artery rings (Dales et al., 2022). It was also reported that the vessel’s response to ATP depends on its initial tone (Neely et al., 1989; Dales et al., 2022). Three different types of vasomotor reaction to ATP have been described in the literature: vasocontraction, rapid relaxation and slow and prolonged vasorelaxation (Ralevic, 2002). This is generally consistent with the previously proposed hypothesis of dual function for ATP in the regulation of vascular tone (Burnstock and Kennedy, 1986). The opposite vascular effects of ATP are mediated by purine P2X- and P2Y-receptors, while different authors provide somewhat conflicting data regarding the involvement of their specific subtypes (Ralevic, 2002; Harrington et al., 2007; Dales et al., 2022; Li et al., 2023).

In cardiological practice, ATP has long been used as an antiarrhythmic agent for the relief of paroxysmal supraventricular tachycardia (Somló, 1955; Saito et al., 1986), including wide QRS complex tachycardia (Sharma et al., 1990). The use of ATP in diagnostics is also well known, for example, for discrimination of monomorphic ventricular tachycardia from aberrant supraventricular tachycardia (Miyoshi et al., 2023), validation of dual atrioventricular nodal physiology (Okumura et al., 2003; Arai et al., 2024), or detection of sick sinus syndrome (Tan et al., 2004). It is noteworthy that experimental and clinical data indicate that ATP can cause both an increase (Boarini et al., 1984; Van Aken et al., 1984; Hussain et al., 2009; Pivovarova et al., 2022; Kong et al., 2023) and decrease in heart rate (Saano et al., 1990; Pelleg and Belhassen, 2010; Pelleg et al., 2012), as well as provoke persistent atrioventricular block (Saano et al., 1990; Kong et al., 2023), or other arrhythmias (Belhassen et al., 1984; Saano et al., 1990; Miyoshi et al., 2023), which indicates the different polarity of the drug’s effect on automatism, excitability, and conductivity of the myocardium. ATP can also be used as a diagnostic agent for the identification of syncope (Pelleg et al., 2012; Fragakis et al., 2015), and as a provocative vasodilating agent in stress perfusion cardiac magnetic resonance imaging for the diagnosis of myocardial ischemia (Kramer et al., 2020; Pivovarova et al., 2022; Kong et al., 2023). These applications once again emphasize its pronounced vasoactive properties. Recently, experimental work has appeared in which ATP was studied as a neutralizer of the damaging effects of various pharmacological substances on organs and tissues. In particular, its protective effect has been demonstrated against 5-Fluorouracil-Induced Oxidative Ovarian Damage (Ozer et al., 2023), bevacizumab-induced kidney damage (Kocaturk et al., 2021), amiodarone-induced optic neuropathy (Bayrakçeken et al., 2023), and cardiotoxicity (Yıldırım et al., 2023). These data contribute to the possible expansion of the scope of clinical use of ATP, which increases the relevance of obtaining additional information about its effect on blood circulation in general and cerebral perfusion in particular.

In our experiments with ATP, we identified pronounced stereotypic changes in cortical perfusion that occurred during about three minutes after the start of drug administration, which we designated as peak, dip, and plateau (see 3.2.1). We found that these changes occur in parallel with the dynamics of system indicators, namely, the peak of APC is achieved at the initial phase of ABP drop and accompanied by a sharp and strong decrease in HR, the dip of APC occurs against the background of extremely low ABP, and the plateau is reached at the initial phase of ABP recovery, which is still remains significantly below the baseline. We hypothesize that the APC peak is due either to relaxation reaction of the cerebral vasculature to the incipient collapse of ABP (an attempt to instantly compensatory increase in brain perfusion in response to a sharp decrease in ABP), or the direct dilative effect of ATP on the intracranial vessels. Of course, a combination of both of these factors is possible. An option of ATP to directly affect cerebral vessels is assumed due to observation of the direct dose dependence of its influence on cerebral perfusion, but not on indicators of systemic hemodynamic (see **Table 3**), and is consistent with the data of other authors (Forrester et al., 1979; Hussain et al., 2009).

The dip of APC has a direct cause-and-effect relationship with the collapsed status, in which extremely low pressure in the vascular network against the background of severe bradycardia is unable to provide adequate local perfusion. This stage is critical for the vital activity of the organism as a whole, which is also evidenced by a significant decrease in end-tidal CO_2_. Such a decrease is consistent with previously published data on the development of severe metabolic acidosis caused by ATP infusion (Boarini et al., 1984). It is worth noting that acidosis itself can be an additional factor in the relaxation of cerebral vessels, as we have previously shown in experiments with dorzolamide (Mamontov et al., 2020b). As for extreme bradycardia, back in 1955 Ernö Somló wrote: “Judging from the E.C.G. changes during the injection, there seems no doubt that the A.T.P. substances arrest the heart action and temporarily cause complete asystole” (Somló, 1955). He calls the condition caused by ATP “mitigated shock” and suggests that this kind of shock therapy allows one to abort paroxysmal tachycardia. To date, it is known that ATP can suppress atrioventricular (AV) nodal conduction and terminate paroxysmal supraventricular tachycardia by creating transient AV block (Miyoshi et al., 2023). At the same time, there is an opinion that the negative chronotropic and dromotropic actions of ATP are mediated by adenosine, the product of ATP’s rapid degradation by ecto-enzymes (it is believed that adenosine depresses AV nodal conduction by direct action on AV nodal cells), and by a cardio-cardiac vagal reflex triggered by ATP’s stimulation of vagal sensory nerve terminals in the left ventricle (Pelleg et al., 2012).

As for the plateau of APC, which is reached against the background of reduced ABP compared to the baseline and almost restored HR, this is an excellent example of a compensatory increase in blood supply to the brain (after a short episode of ischemia) due to an emergency redistribution of blood flow in favor of the vital organ and indicates the presence of a significant cerebrovascular reserve. Comparing the dynamics of APC and ABP at the time points of the peak and plateau, we conclude that these indicators vary over time in different directions, since APC increases with a decrease in ABP. This relationship is understandable from the point of view of the need to maintain brain perfusion. However, this rule stops working with a catastrophic collapse of ABP, which is observed at the dip point of APC, when, due to transient cardiovascular failure, the systemic ABP is so low that adequate organ blood supply cannot be ensured in principle.

Therefore, we have demonstrated in our experiments with ATP that the APC signal from intracranial tissues differentially relates to the ABP level: in the collapsed status, APC decreases, signaling a critical situation with the blood supply to the brain. However, APC increases significantly with a more or less compensated decrease in ABP, and it does not matter whether it further decreases or increases, which means that an adaptive mechanism is activated to maintain high cerebral perfusion in conditions of relative insufficiency of systemic hemodynamic. These data confirm our earlier assumption that APC is a kind of surrogate of local vasodilation since its increase is due to a decrease in arterial tone in biological tissues in the area of registration of IPPG signals (Lyubashina et al., 2019; Mamontov et al., 2020b; Volynsky et al., 2022; Sokolov et al., 2023). Moreover, our experiments confirmed the vasodilating, hypotensive and cardio-depressant properties of ATP, demonstrated the safety of the drug when administered systemically, and showed the possibility of its use in provocative pharmacological tests.

More recently, our group carried out a direct experimental comparison of IPPG and Laser Speckle Contrast Imaging (LSCI) in rats to evaluate changes in cerebral perfusion caused by the ATP administration, using both modalities simultaneously (Kamshilin et al., 2025). It has been shown that the dynamics of changes in blood flow velocity, assessed by the LSCI system using a generally accepted data processing algorithm, radically differs from the dynamics of the perfusion index, estimated by the IPPG system. However, when the LSCI data was processed using new algorithm proposed in (Kamshilin et al., 2025), the dynamics of the circulatory response to a vasoactive agent became similar to the perfusion dynamics assessed by IPPG. It is worth noting that the new LSCI data processing algorithm is essentially the same as for IPPG data: it is based on calculating the amplitude of speckle contrast modulation in each cardiac cycle (i.e., pulsatile modulation), thereby allowing the assessment of the cortical perfusion index. The fundamental difference between LSCI and IPPG is that the main cause for signal modulation in the former is light scattering, whereas in the latter it is light absorption. Consequently, their combined use in monitoring changes in blood supply might provide a comprehensive assessment of the physiological causes of hemodynamic changes (Kamshilin et al., 2025).

Norepinephrine (NE) is a monoamine neurotransmitter and precursor of epinephrine, participates in a variety of biological processes and is involved in the pathophysiology of a number of nosologies, in particular, neurodegenerative conditions (Holland et al., 2021; Portela Moreira et al., 2023), cardiovascular diseases (Marques et al., 2017; Wood and Valentino, 2017), major depressive disorder (Moriguchi et al., 2017), chronic inflammatory diseases (Sohn and Jenei-Lanzl, 2023), headaches (Sokolov et al., 2013), attention-deficit/hyperactivity disorder (del Campo et al., 2011), etc. Along with epinephrine, dobutamine and dopamine, NE is widely used in intensive care units as a cardio- and vasotonic agent for the purpose of catecholamine support of hemodynamic; in some types of shock, NE is deservedly considered as a first-line treatment drug (Annane et al., 2018; Jentzer and Hollenberg, 2021; Russell et al., 2021).

In our experiments with NE, we also identified stereotypical changes in the APC signal that occurred during about four minutes after the start of drug administration, which were designated as peak, dip, and plateau. As with ATP infusion, these changes correlate with certain fluctuations in systemic hemodynamic (see Sect. 3), namely: the APC peak corresponds to a powerful hypertensive surge, the dip occurs against the background of trace hypertension, and plateau is reached at a significantly lower ABP compared to the baseline. We suggest the following explanation of the observed peculiarities. Since NE is a strong vasoconstrictor, it apparently causes an avalanche-like crisis rise in ABP after bolus administration in a high dose. Such a jump in ABP is accompanied by a sharp non-physiological increase in brain perfusion: adaptive vascular mechanisms, which maintain constant cerebral perfusion and protect the brain from blood overload (oedema) and possible damage (Tzeng and Ainslie, 2014; Shekhar et al., 2017; Silverman and Petersen, 2024), simply do not have time to work or their power is insufficient at such a high rate and degree of pressure increase. In turn, the dip, characterized by significantly less magnitude of APC compared to the baseline, is observed about 40 s after the peak, when ABP remains at a level 1.5 times higher than the baseline. This fact precisely indicates the successful, albeit somewhat delayed, activation of these mechanisms of cerebrovascular adaptation: at high ABP, the brain is protected from damaging hyperperfusion due to vasoconstriction, which is expressed as a decrease in APC. Finally, at the plateau, APC again becomes more than twice as high as compared to the baseline, but ABP drops significantly below the baseline. This means developing a situation with low blood pressure: cerebrovascular tone also decreases, which is reflected by an increase in APC. It is worth noting that the trend of increased APC against the background of relatively low blood pressure persists until the end of the session, which indicates stable maintenance of cerebral perfusion against the background of rebound hypotension.

The administration of NE had a relatively weak effect on HR (only at the dip point an episode of tachycardia occurred) and was accompanied by a statistically significant transient increase in end-tidal CO_2_, which is generally explained by the pharmacodynamics of the drug.

Therefore, in our experiments with NE, we confirmed the hypothesis stated above about the differentiated relationship between APC and ABP and demonstrated patterns of changes in brain perfusion during a high-dose bolus load of the drug. Continuing the discussion about the cerebrovascular response to norepinephrine raised by (Froese et al., 2020), we can add that, according to our current study, NE has a dual effect on cerebral vasculature and perfusion. Consequently, in each specific case, provided there is no hypovolemia, it is possible to select a dose and rate of administration that will maintain not only ABP, but also brain perfusion at an optimal level.

An important limitation of our study is that the APC parameter does not provide an absolute measure of perfusion. It is just a relative index of perfusion, which can vary greatly from one type of biological tissue to another, as well as in different experimental animals or subjects. However, when comparing APC at the same study points before, during, or after the pharmacological test, conclusions can be drawn about the nature of the restructuring of the regional blood supply based on the measured APC dynamics. Methodologically, the APC reflects the dynamics of changes in the tone of arteries supplying the area under study if the condition of stable illumination of the measured area of tissue and compensation for its mechanical movement relative to the light source are fulfilled.

## 5 Conclusions

Despite the extensive use of IPPG as a helpful tool for carrying out clinical and experimental studies and diagnostics, the nature of IPPG signal formation remains the subject of debate, the key point of which is the discussion of the relationship of local vascular reactions (assessed by changes in APC) with fluctuations in the parameters of systemic hemodynamics. Our experiments have demonstrated that IPPG is a contactless method that allows us to quantify changes in cortical perfusion with high sensitivity and high resolution in both space and time. The APC parameter is a surrogate for the brain tissue perfusion index, which makes it possible to monitor changes in perfusion to both the cerebral cortex as a whole and its individual lobes. Using the well-known pharmacological substances NE and ATP as provocateurs of dramatic multidirectional changes in blood pressure, we were able to show and explain for the first time a clear relationship between systemic and intracranial hemodynamic changes. These observations allowed us to substantiate that the contribution to changes in APC is provided not only by superficial changes in microcirculation, but also by vascular events located deeper in the tissue under study, which in our current study was the brain. Moreover, the data obtained in this study expand the understanding of the pharmacodynamics of NE and ATP in terms of their cerebrovascular effects, which is important in light of their widespread use in practical medicine as therapeutic and diagnostic drugs.

## Data Availability

The raw data supporting the conclusions of this article will be made available by the authors, without undue reservation.
