# Supplementary material for: Imaging photoplethysmography reveals differences in the reactions of cerebral and systemic hemodynamics to infusion of vasoactive drugs
Source: Front Physiol. 2026 Mar 19;17:1777457. doi: 10.3389/fphys.2026.1777457 (PMC13043429; doi:10.3389/fphys.2026.1777457)
Supplement: Supplementary file 1 [file DataSheet1.pdf]

Supplementary Material

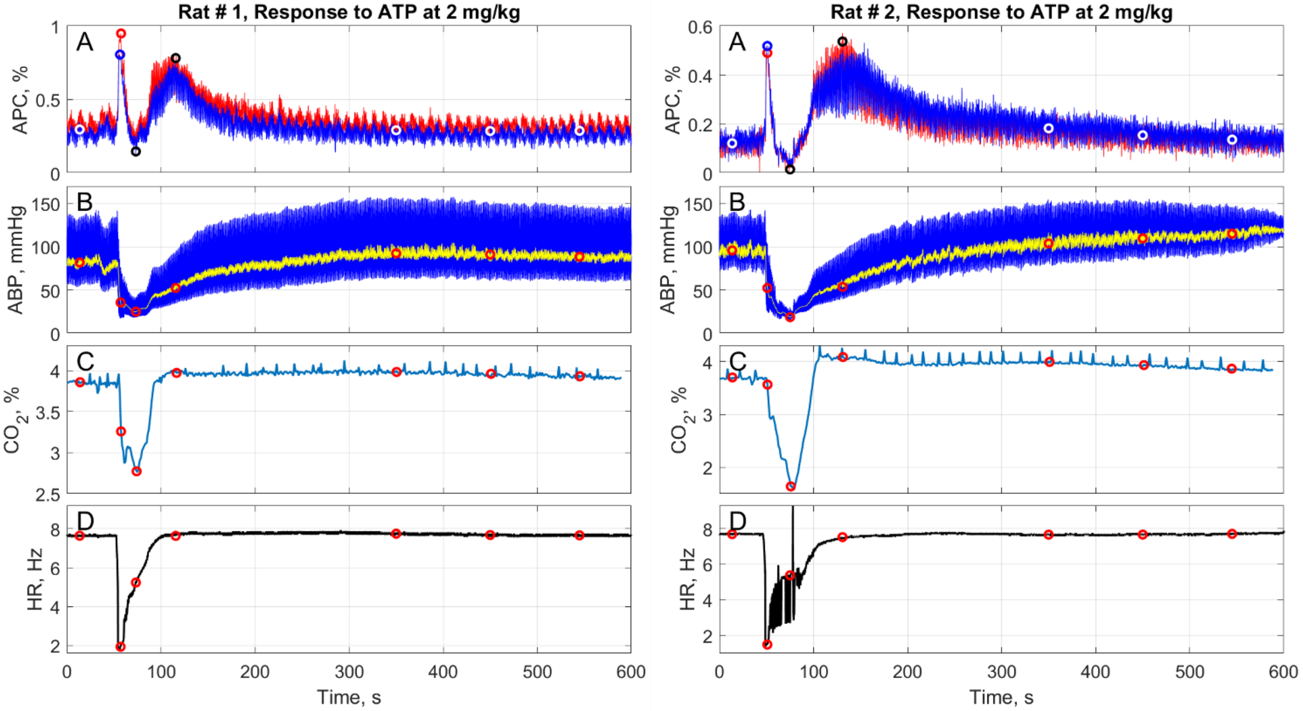

Supplementary Figure S1. Responses of cortical and systemic blood flow parameters in rats No.1 and No.2 to infusion of adenosine triphosphate at a dose of 2 mg/kg.

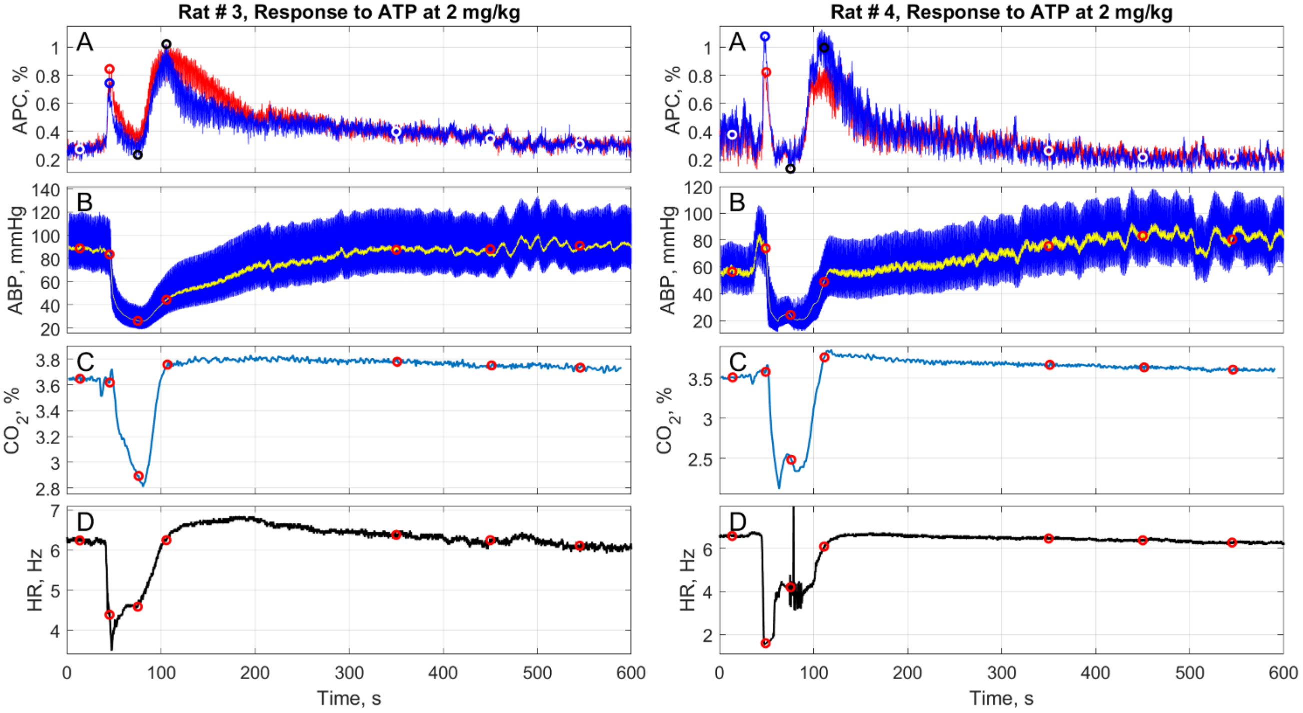

Supplementary Figure S2. Responses of cortical and systemic blood flow parameters in rats No.3 and No.4 to infusion of adenosine triphosphate at a dose of 2 mg/kg.

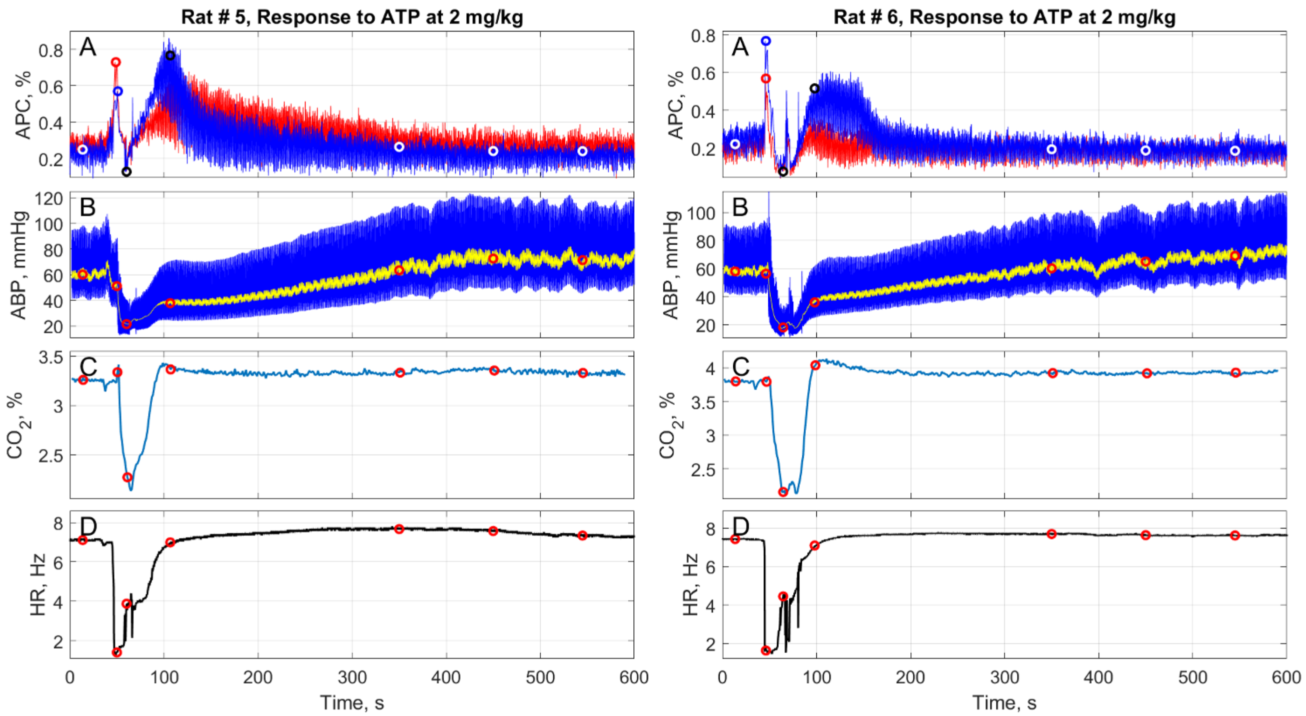

Supplementary Figure S3. Responses of cortical and systemic blood flow parameters in rats No.5 and No.6 to infusion of adenosine triphosphate at a dose of 2 mg/kg.

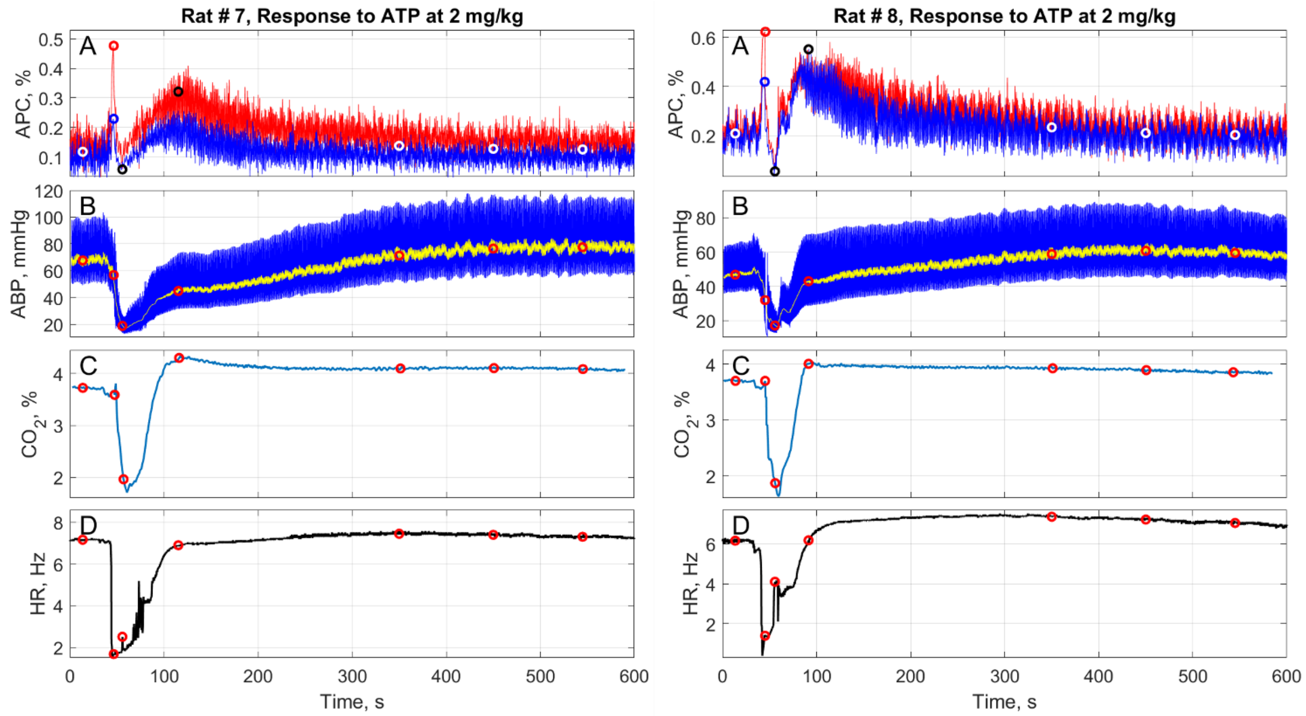

Supplementary Figure S4. Responses of cortical and systemic blood flow parameters in rats No.7 and No.8 to infusion of adenosine triphosphate at a dose of 2 mg/kg.

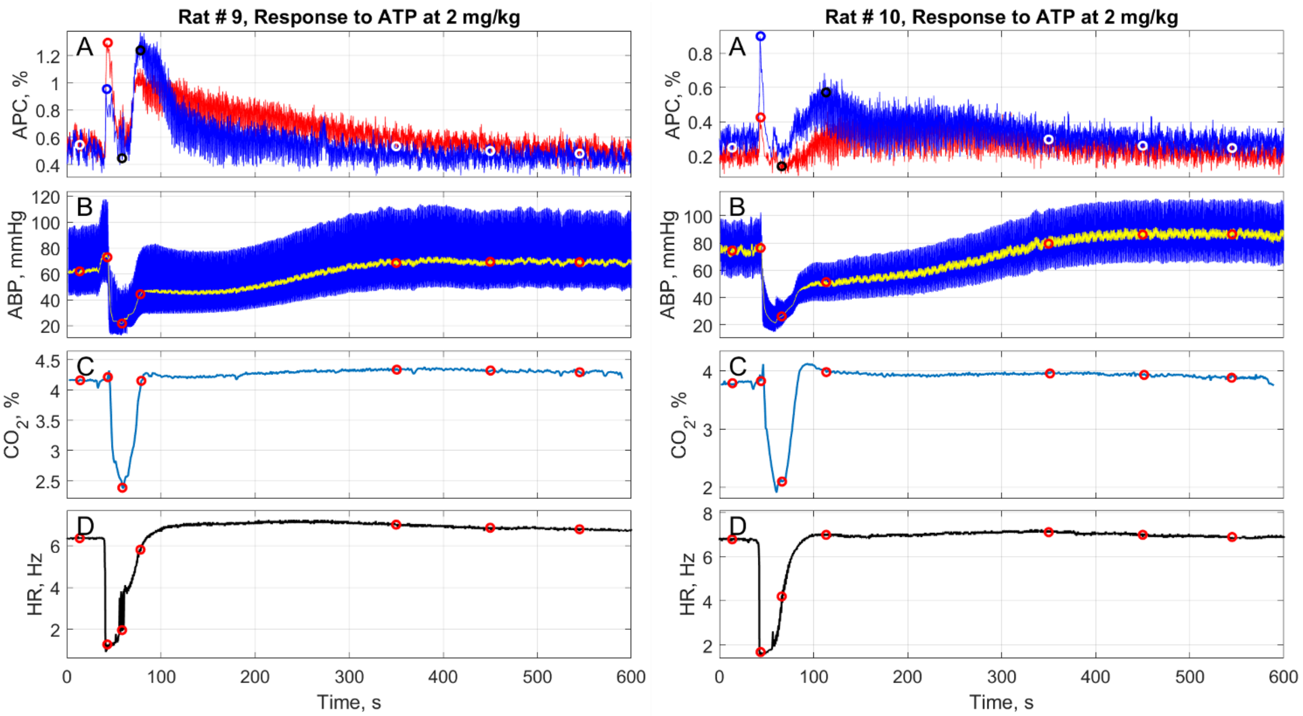

Supplementary Figure S5. Responses of cortical and systemic blood flow parameters in rats No.9 and No.10 to infusion of adenosine triphosphate at a dose of 2 mg/kg.

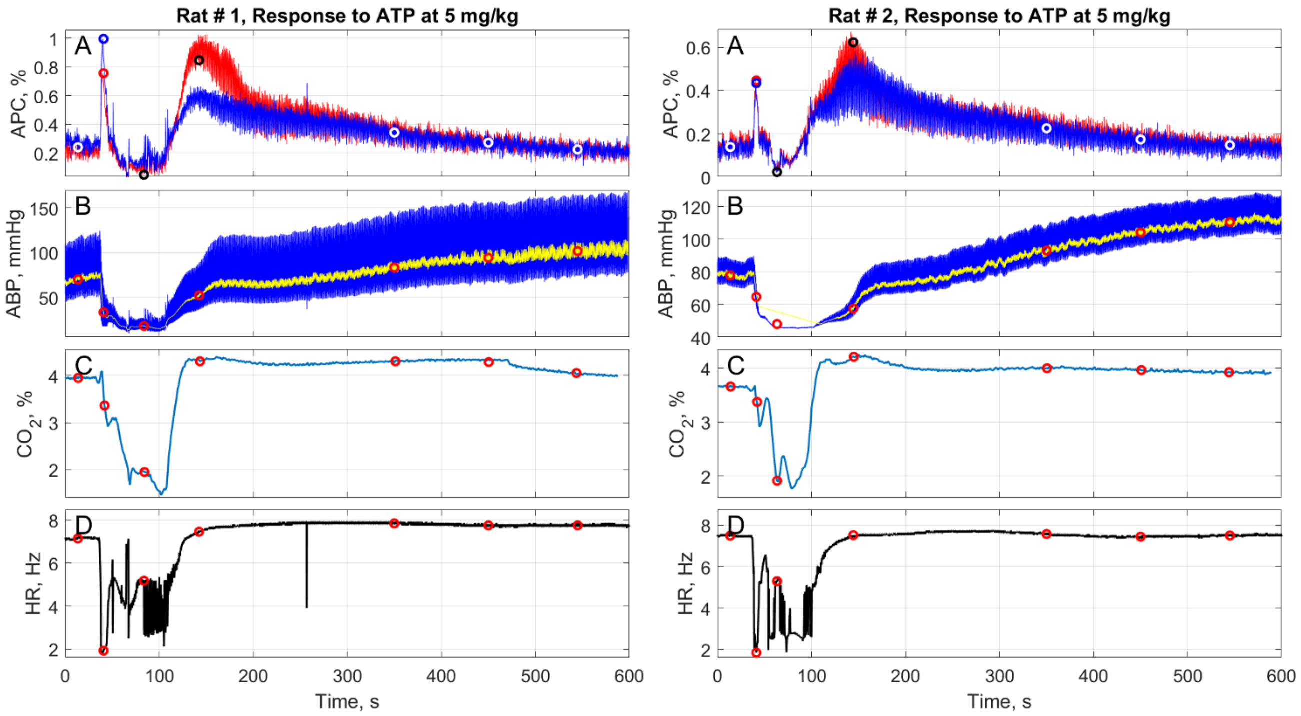

Supplementary Figure S6. Responses of cortical and systemic blood flow parameters in rats No.1 and No.2 to infusion of adenosine triphosphate at a dose of 5 mg/kg.

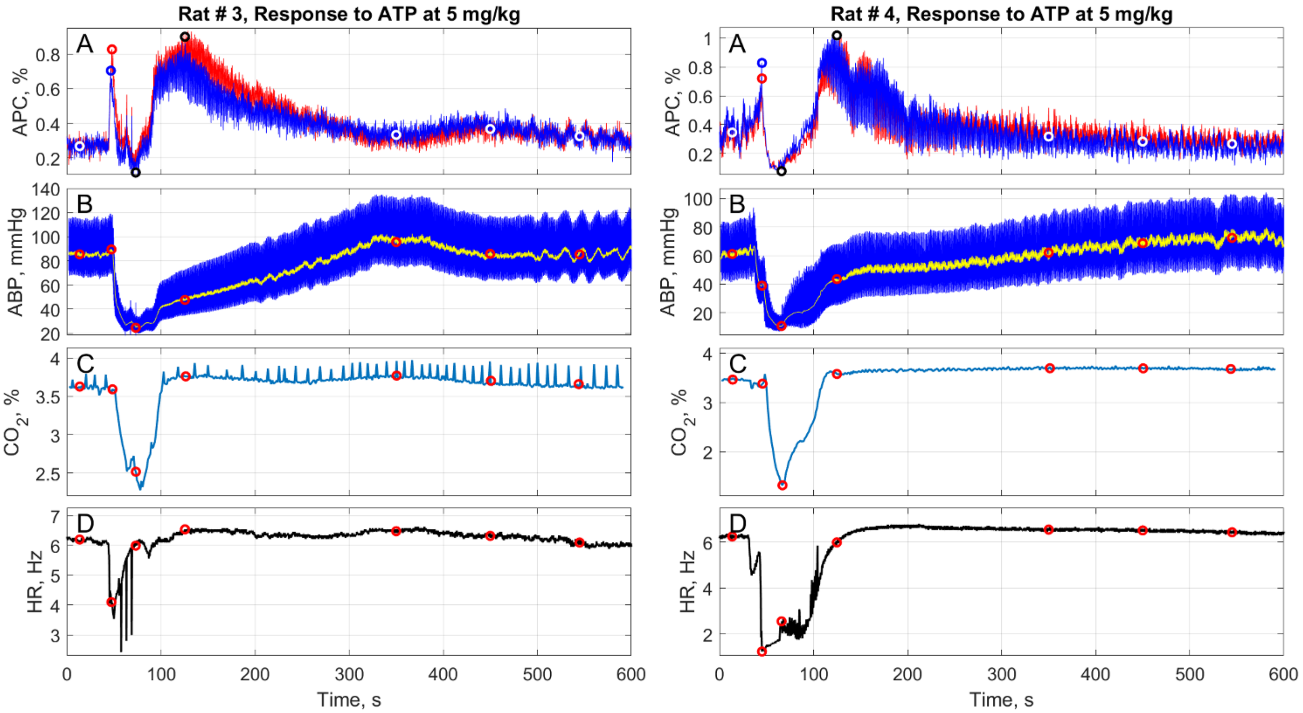

Supplementary Figure S7. Responses of cortical and systemic blood flow parameters in rats No.3 and No.4 to infusion of adenosine triphosphate at a dose of 5 mg/kg.

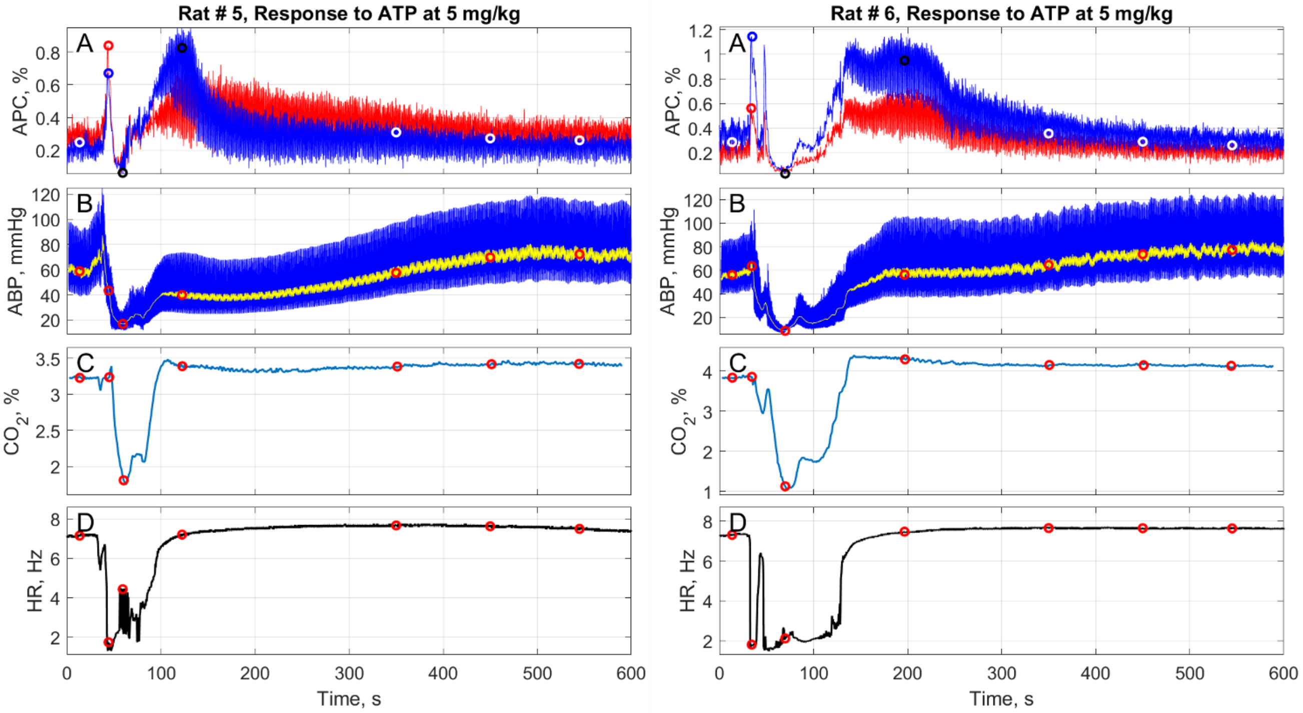

Supplementary Figure S8. Responses of cortical and systemic blood flow parameters in rats No.5 and No.6 to infusion of adenosine triphosphate at a dose of 5 mg/kg.

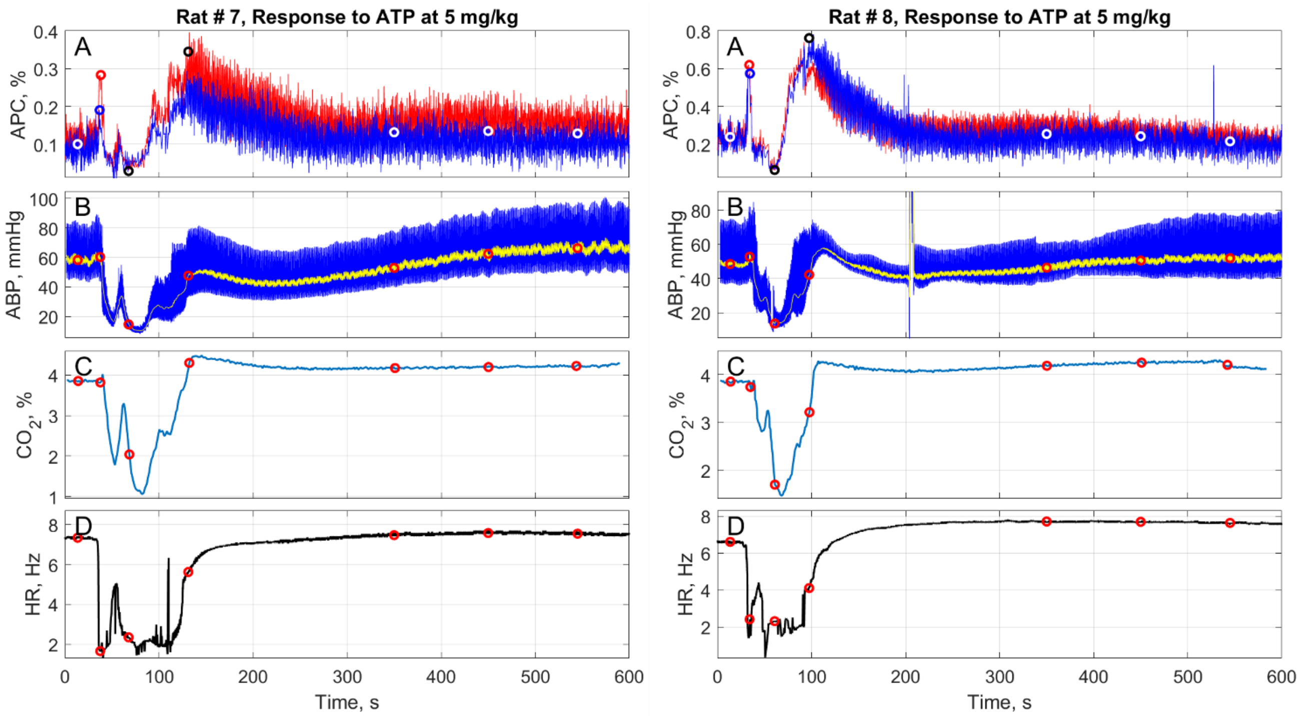

Supplementary Figure S9. Responses of cortical and systemic blood flow parameters in rats No.7 and No.8 to infusion of adenosine triphosphate at a dose of 5 mg/kg.

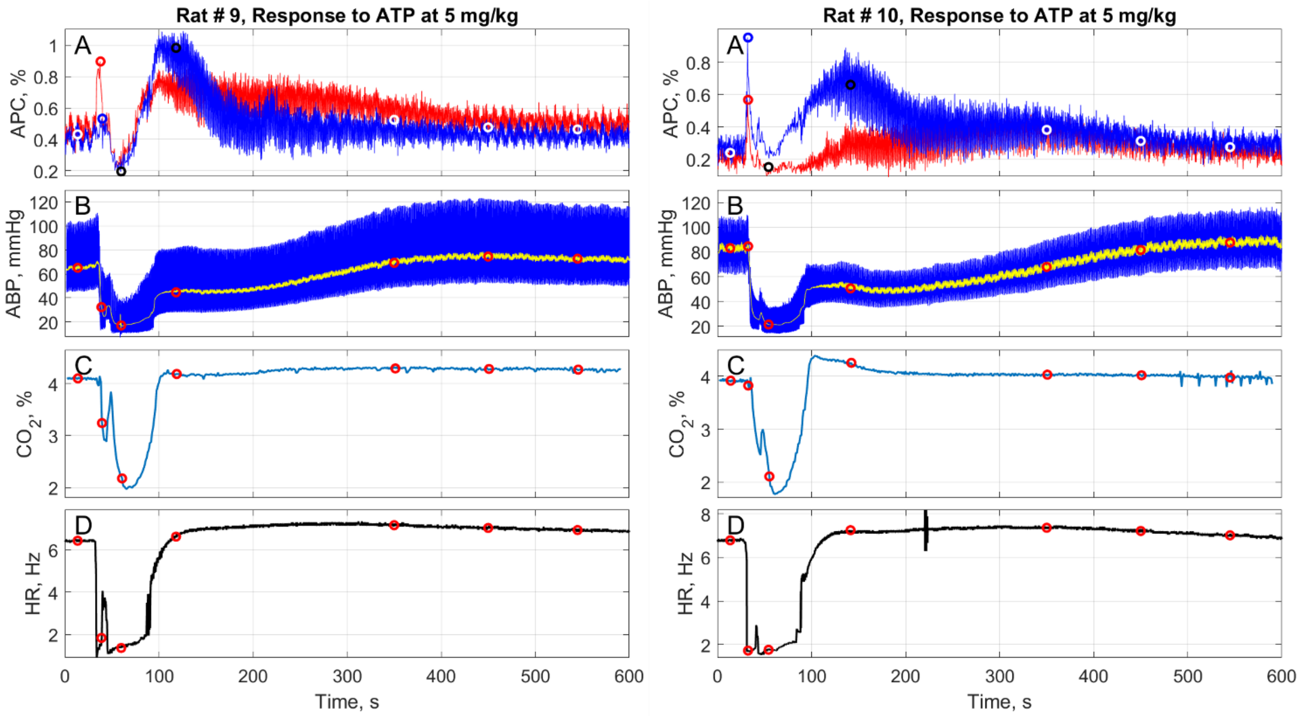

Supplementary Figure S10. Responses of cortical and systemic blood flow parameters in rats No.9 and No.10 to infusion of adenosine triphosphate at a dose of 5 mg/kg.

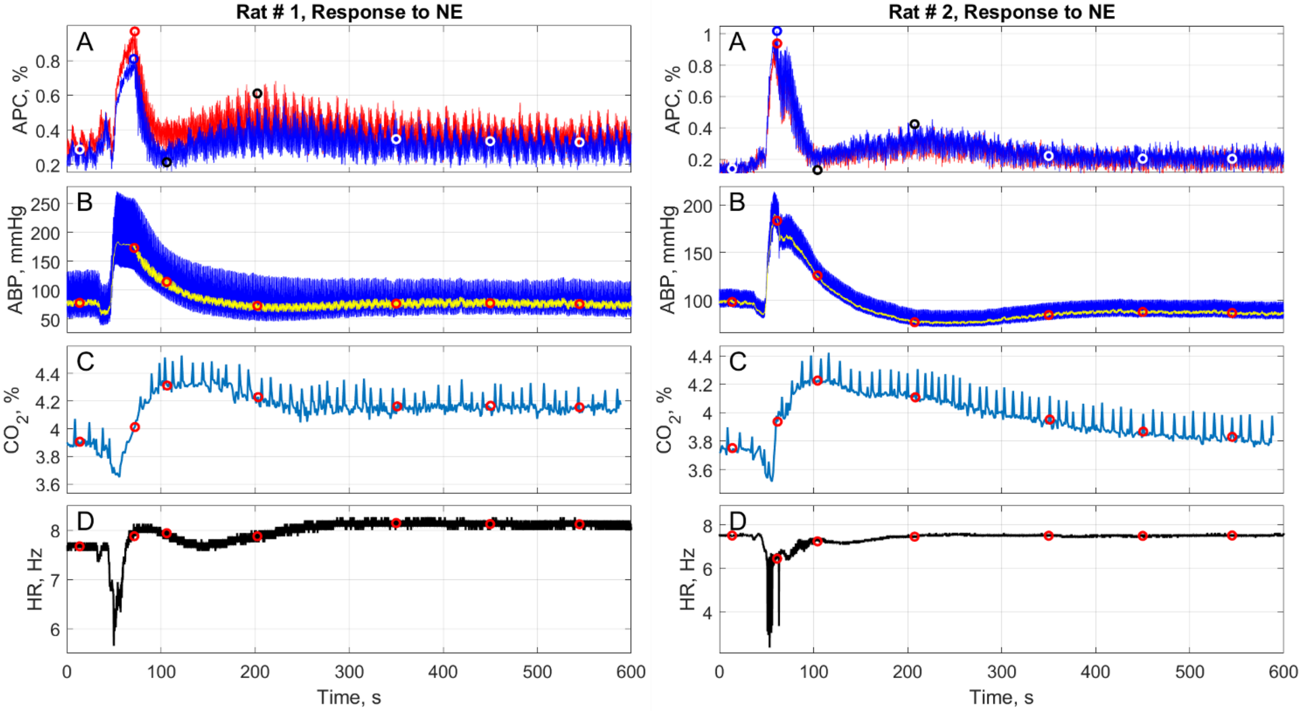

Supplementary Figure S11. Responses of cortical and systemic blood flow parameters in rats No.1 and No.2 to infusion of norepinephrine at a dose of 10 μg/kg.

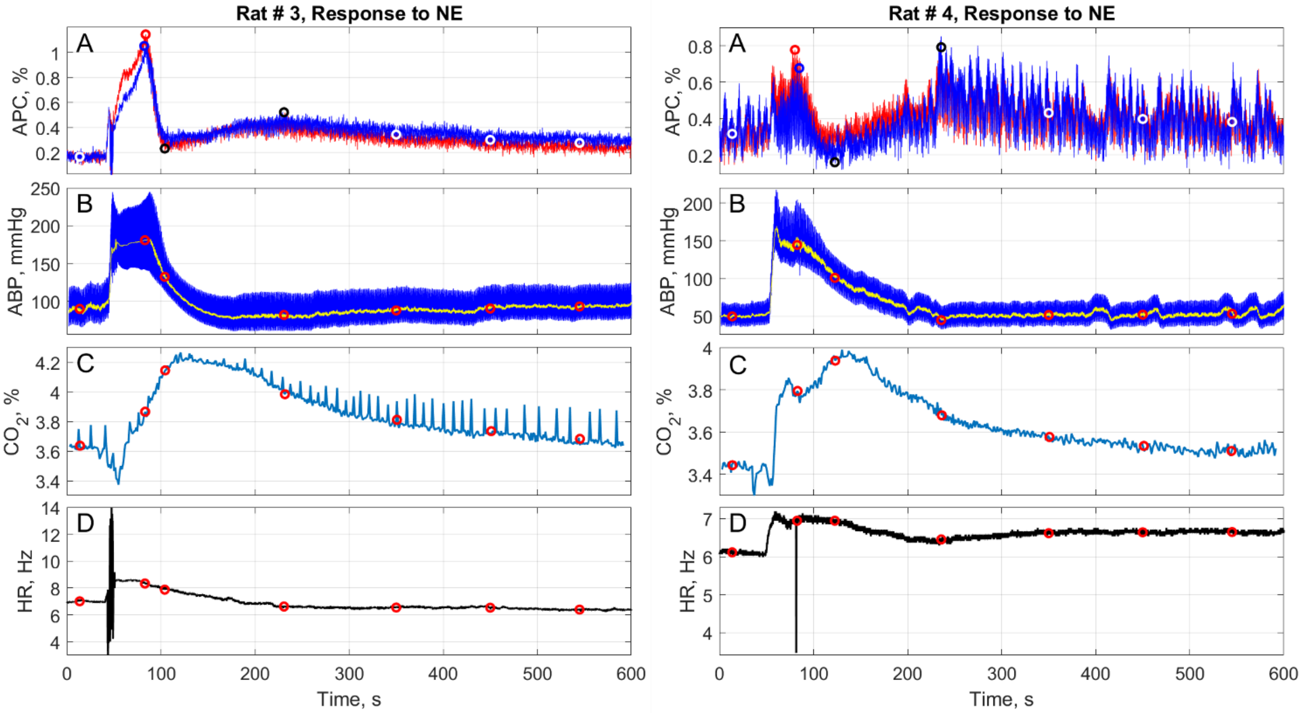

Supplementary Figure S12. Responses of cortical and systemic blood flow parameters in rats No.3 and No.4 to infusion of norepinephrine at a dose of 10 μg/kg.

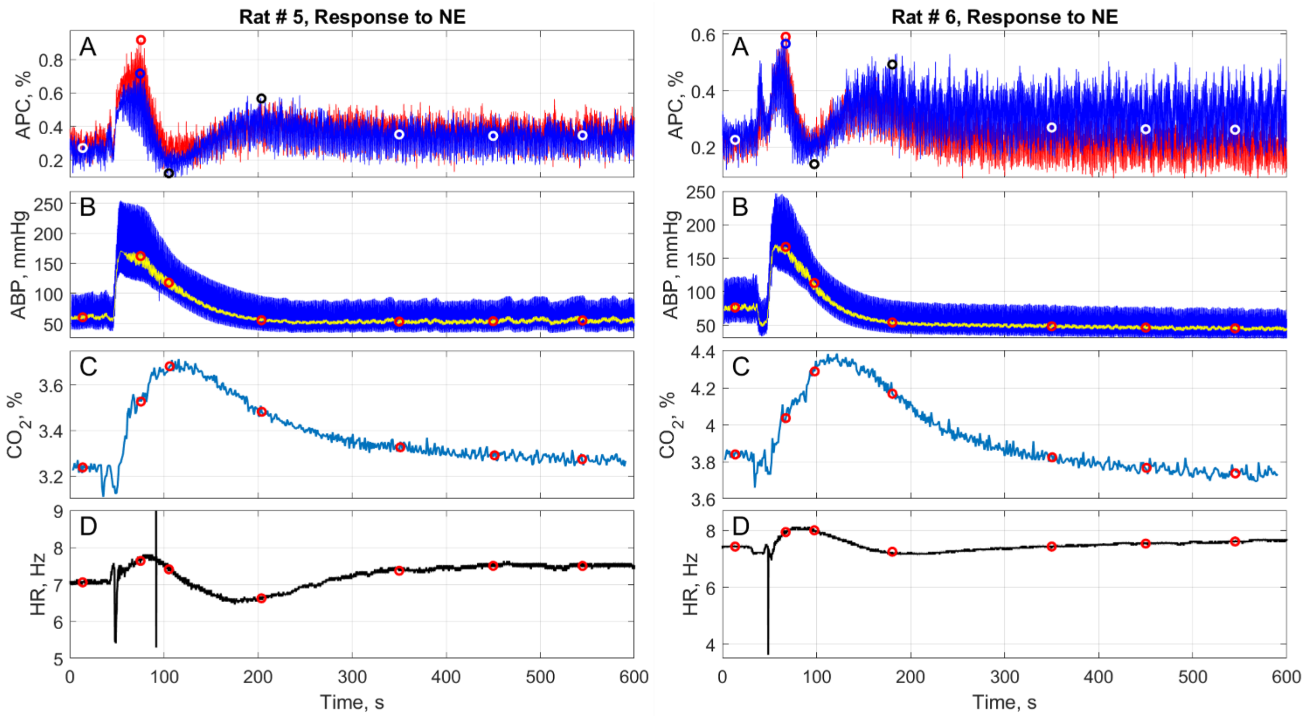

Supplementary Figure S13. Responses of cortical and systemic blood flow parameters in rats No.5 and No.6 to infusion of norepinephrine at a dose of 10 μg/kg.

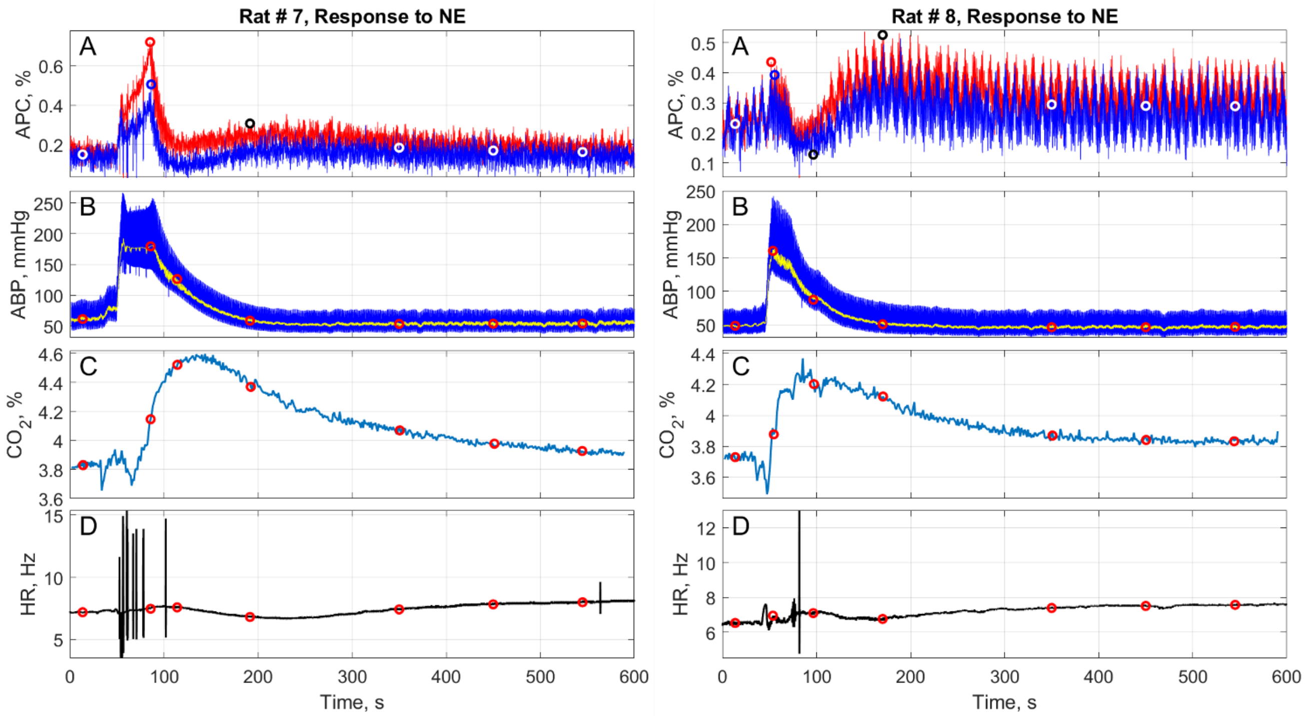

Supplementary Figure S14. Responses of cortical and systemic blood flow parameters in rats No.7 and No.8 to infusion of norepinephrine at a dose of 10 μg/kg.

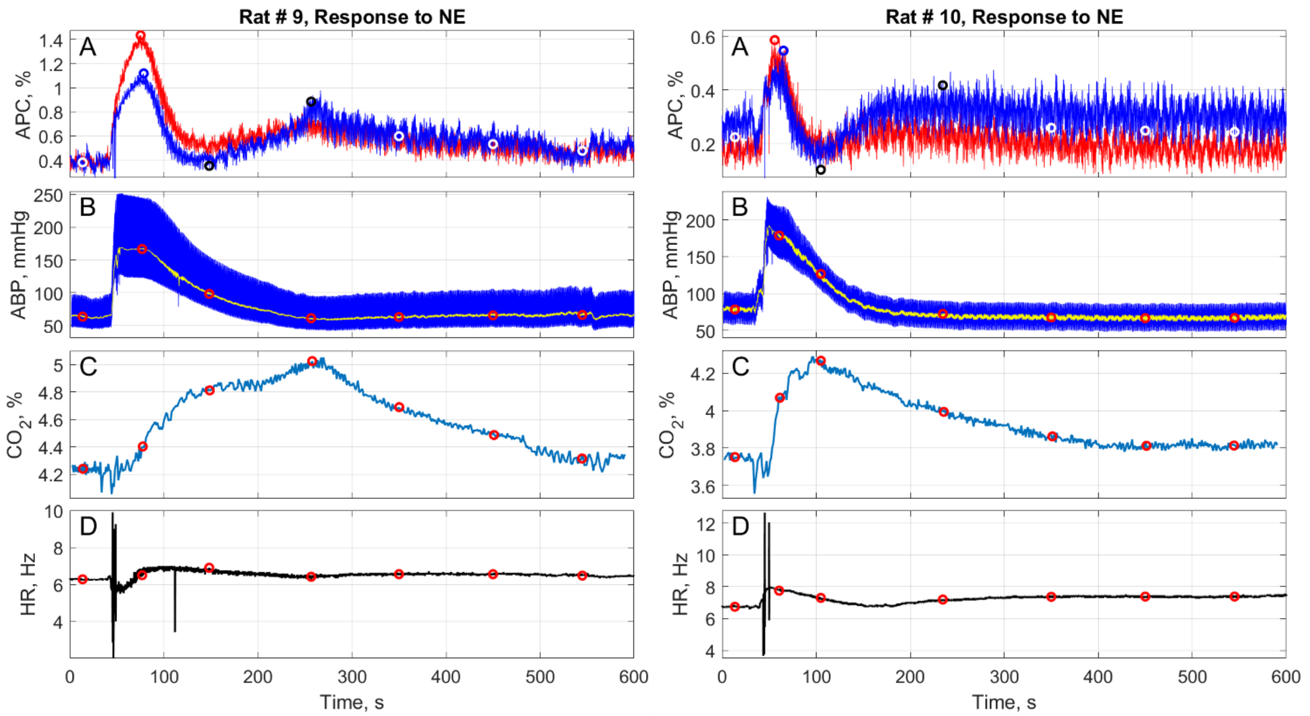

Supplementary Figure S15. Responses of cortical and systemic blood flow parameters in rats No.9 and No.10 to infusion of norepinephrine at a dose of 10 µg/kg.

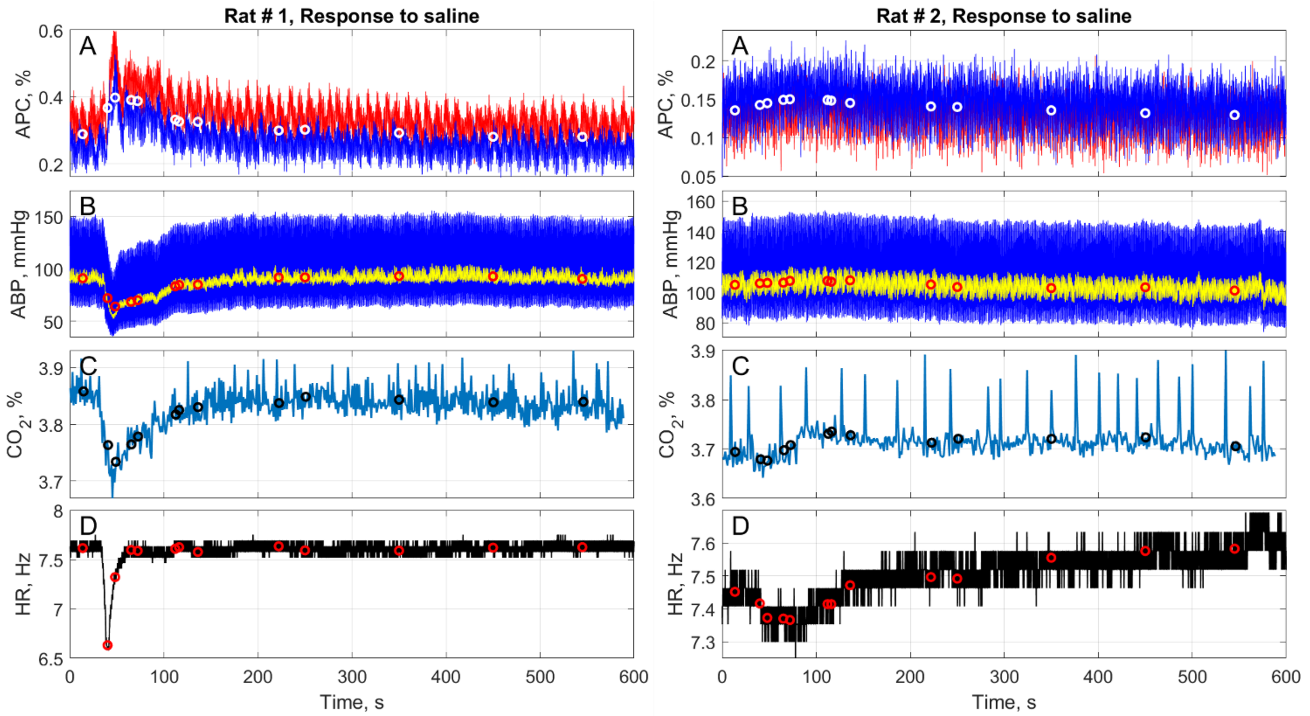

Supplementary Figure S16. Responses of cortical and systemic blood flow parameters in rats No.1 and No.2 to saline infusion.

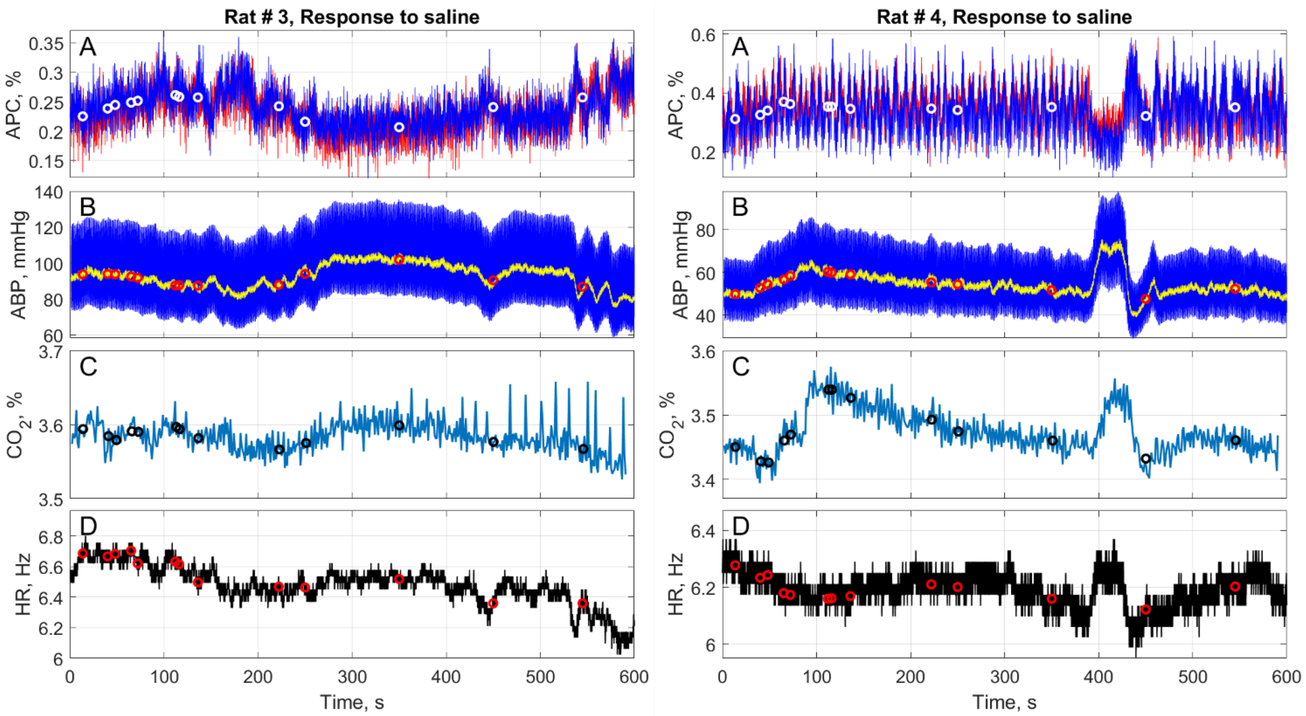

Supplementary Figure S17. Responses of cortical and systemic blood flow parameters in rats No.3 and No.4 to saline infusion.

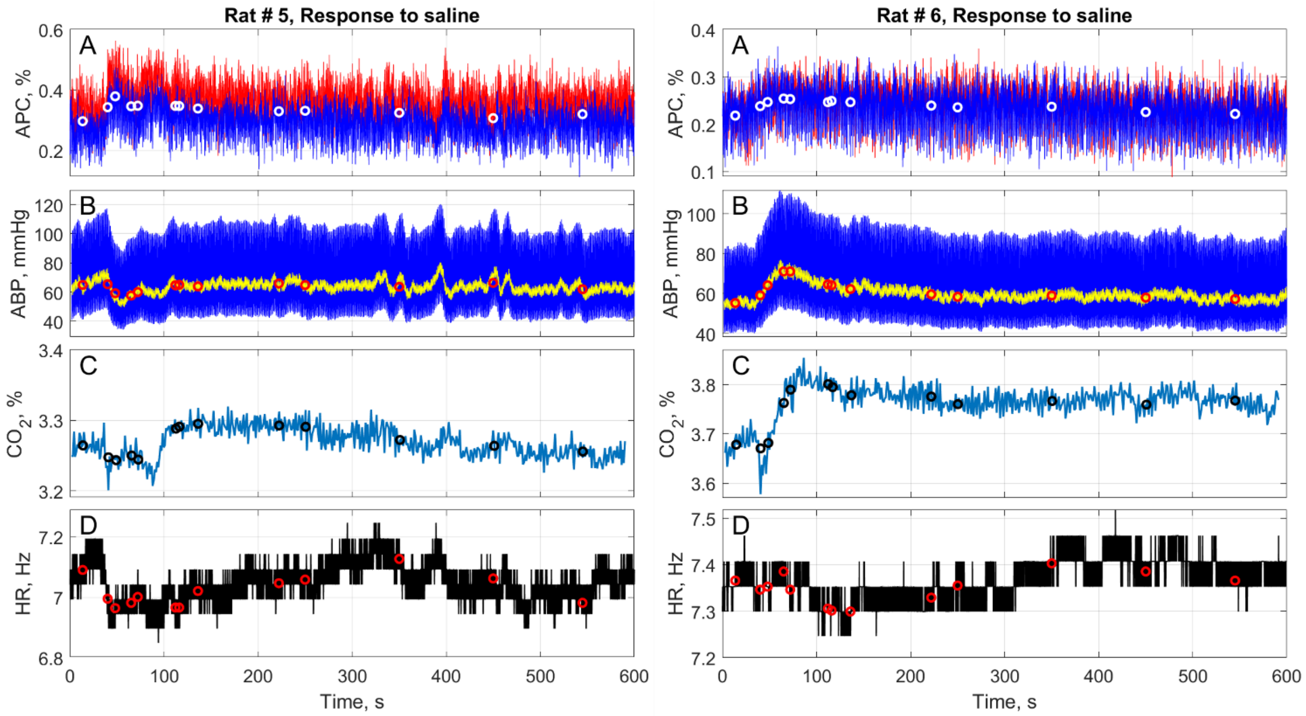

Supplementary Figure S18. Responses of cortical and systemic blood flow parameters in rats No.5 and No.6 to saline infusion.

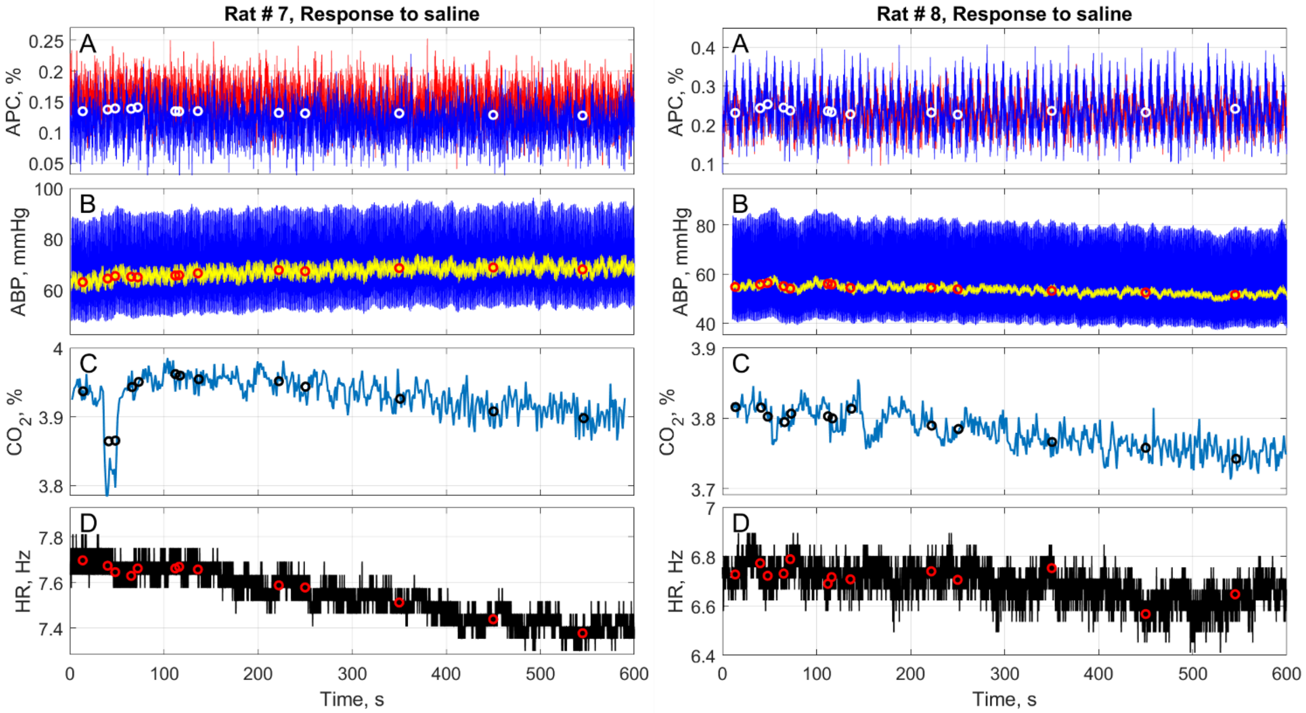

Supplementary Figure S19. Responses of cortical and systemic blood flow parameters in rats No.7 and No.8 to saline infusion.

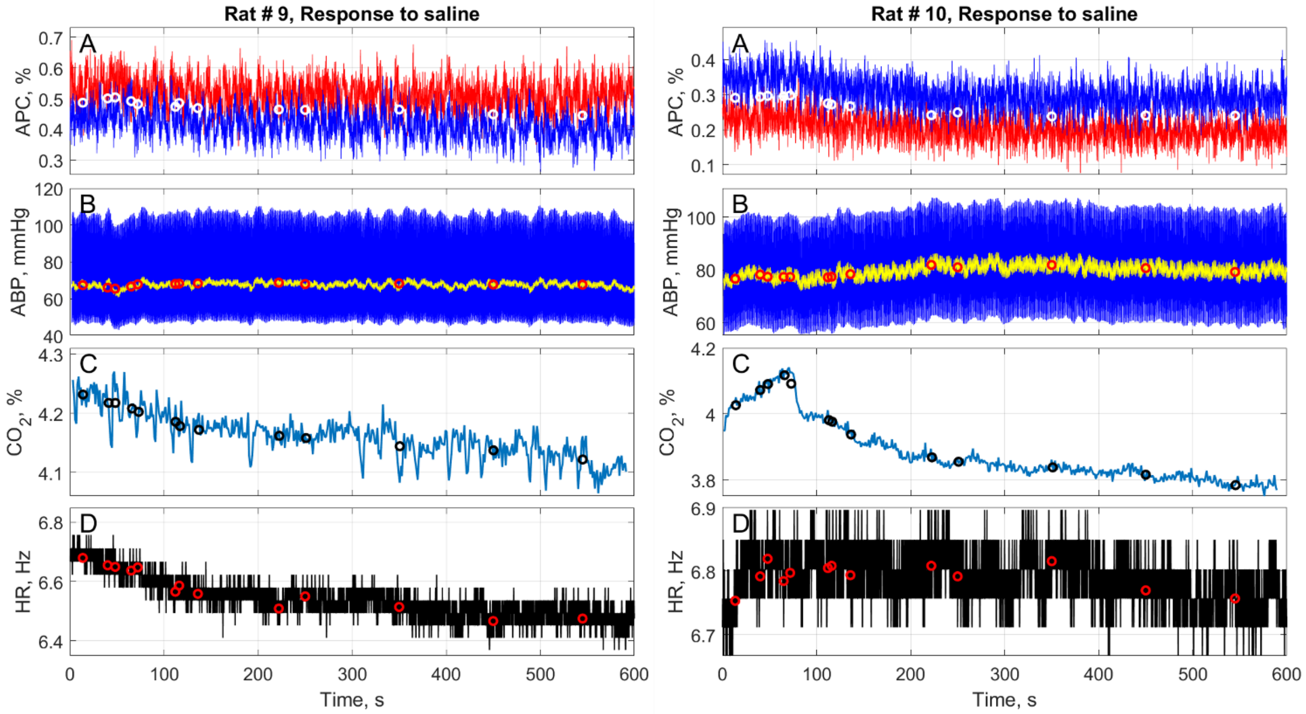

Supplementary Figure S20. Responses of cortical and systemic blood flow parameters in rats No.9 and No.10 to saline infusion.
